# Supplementary material for: Paediatric oncology nursing education and training programmes: a scoping review protocol
Source: BMJ Open. 2023 Oct 9;13(10):e070694. doi: 10.1136/bmjopen-2022-070694 (PMC10565288; doi:10.1136/bmjopen-2022-070694)
Supplement: Supplementary data [file bmjopen-2022-070694supp001.pdf]

Appendix 1: Search Strategies used across databases

Database	Items Searched	Results
Scopus	1. TITLE-ABS-KEY ("continuous professional development" OR "short learning programme" OR "in-service training" OR "nursing education" OR "service training")	118,479
	2. neoplasm* OR "paediatric cancer" OR "childhood cancer"	3,185,488
	3. TITLE-ABS-KEY ("pediatric oncology nursing" OR "paediatric oncology" OR "oncology nursing" OR "paediatric oncology nursing")	17,449
	4. (TITLE-ABS-KEY ("continuous professional development" OR "short learning programme" OR "in-service training" OR "nursing education" OR "service training")) AND (neoplasm OR "paediatric cancer'" OR "childhood cancer") AND (TITLE-ABS-KEY ("pediatric oncology nursing" OR "paediatric oncology" OR "oncology nursing" OR "paediatric oncology nursing")	**152**
PubMed	1. ((((neoplasm [MeSH Terms]) OR ('paediatric cancer'[Title/Abstract])) OR ('childhood cancer'[Title/Abstract])	1,271,317
	2. ((((pediatric oncology nursing [Title/Abstract]) OR (Paediatric oncology [Title/Abstract])) OR (oncology nursing [MeSH Terms])) OR (paediatric oncology nursing [Title/Abstract])	3082
	3. ((((continuous professional development [Title/Abstract]) OR (short learning programme [Title/Abstract])) OR (Inservice Training [MeSH Terms])) OR (education, nursing [MeSH Terms]	26,276
	4. #1 AND #2 AND #3	**102**
Embase	1. 'neoplasm'/exp OR neoplasm OR 'paediatric cancer' OR 'childhood cancer'/exp OR 'childhood cancer'	6,021,304
	2. 'pediatric oncology nursing'	973
	3. 'nursing education' OR 'in service training' OR 'continuing education' OR 'continuous professional development'	148,334
	4. #1 AND #2 AND #3	**92**
Dimensions	1. "Paediatric oncology" OR "Paediatric oncology nursing" AND "childhood cancer" OR "Paediatric	**36**

Majamanda MD, *et al. BMJ Open* 2023; 13:e070694. doi: 10.1136/bmjopen-2022-070694

	cancer" OR neoplasms AND "nursing education" OR "in service training" OR "continuing education" OR "continuous professional development"	
CINAHL	1. SU neoplasms OR SU paediatric cancer OR SU childhood cancer OR SU paediatric oncology	1,007
	2. TX pediatric oncology nursing OR TX paediatric oncology OR TX oncology nursing OR TX pediatric oncology nursing	138
	3. SU continuous professional development OR TX short learning program* OR TX in-service training OR TX in service training for nurses OR SU nursing education OR TX service training	1854
	4. S1 AND S2 AND S3	**1216**
St. Jude Children's Research Hospital	1. paediatric oncology nursing OR childhood cancer nursing AND continuing education OR orientation training OR educational programmes OR in-service training	**916**

Majamanda MD, *et al. BMJ Open* 2023; 13:e070694. doi: 10.1136/bmjopen-2022-070694
