# Supplementary material for: Paediatric oncology nursing education and training programmes: a scoping review protocol
Source: BMJ Open. 2023 Oct 9;13(10):e070694. doi: 10.1136/bmjopen-2022-070694 (PMC10565288; doi:10.1136/bmjopen-2022-070694)
Supplement: Supplementary data [file bmjopen-2022-070694supp002.pdf]

Appendix 2: A draft data extraction tool

	**Studies**			
**Item**	**1**	**2**	**3**	**4 etc.**
Author				
Year				
Journal				
Country				
Host site/ institution				
Collaborations				
Partner country				
Partner institution				
Location of training				
Project title				
Target population				
Theory content				
Practical areas				
Programme delivery methods				
Programme duration				
Mode of assessment				
Cultural/ contextual issues				
Theoretical framework				
