# Supplementary material for: Paediatric oncology nursing education and training programmes: a scoping review protocol
Source: BMJ Open. 2023 Oct 9;13(10):e070694. doi: 10.1136/bmjopen-2022-070694 (PMC10565288; doi:10.1136/bmjopen-2022-070694)
Supplement: Author's manuscript [file bmjopen-2022-070694.draft_revisions.pdf]

BMJ Open is committed to open peer review. As part of this commitment we make the peer review history of every article we publish publicly available.

When an article is published we post the peer reviewers' comments and the authors' responses online. We also post the versions of the paper that were used during peer review. These are the versions that the peer review comments apply to.

The versions of the paper that follow are the versions that were submitted during the peer review process. They are not the versions of record or the final published versions. They should not be cited or distributed as the published version of this manuscript.

BMJ Open is an open access journal and the full, final, typeset and author-corrected version of record of the manuscript is available on our site with no access controls, subscription charges or pay-per-view fees (http://bmjopen.bmj.com).

If you have any questions on BMJ Open's open peer review process please email info.bmjopen@bmj.com

BMJ Open

Paediatric oncology nursing education and training programs: A scoping review protocol

Journal:	*BMJ Open*
Manuscript ID	bmjopen-2022-070694
Article Type:	Protocol
Date Submitted by the Author:	07-Dec-2022
Complete List of Authors:	Majamanda, Maureen; Kamuzu University of Health Sciences, Child Health Nursing<br>Chisoni, Felix; Kamuzu University of Health Sciences, Library<br>Selemani, Apatsa; Kamuzu University of Health Sciences, Library<br>Kearns, Irene; University of the Witwatersrand Johannesburg Faculty of Health Sciences, Nursing Education<br>Maree, Johanna; University of the Witwatersrand Johannesburg, Nursing Education
Keywords:	ONCOLOGY, Paediatric oncology < ONCOLOGY, PUBLIC HEALTH

SCHOLARONE™
Manuscripts

Title:

Paediatric oncology nursing education and training programs: A scoping review protocol

AUTHORS:

Maureen D. Majamanda[1,2,3], Felix Chisoni[4], Apatsa Selemani [2,4,5], Irene Kearns[3], Johanna E. Maree[3]

Corresponding author

Maureen D. Majamanda, Kamuzu University of Health Sciences, School of Nursing, Chipatala Avenue, P.O Box 415, Blantyre, Malawi.

Email: mdmajamanda@kuhes.ac.mw

Cell phone: +265 (0) 992 160 415

Fax: +(265) 018753941

Co-authors

Felix Chisoni, Kamuzu University of Health Sciences, Blantyre, Malawi.

Apatsa Seleani, Kamuzu University of Health Sciences, Blantyre, Malawi.

Irene Kearns, University of the Witwatersrand, Johannesburg, South Africa.

Johanna E. Maree, University of the Witwatersrand, Johannesburg, South Africa.

Key words

Paediatric oncology nursing, continuing education, orientation training, educational programmes, in-service training

Word count

2709

For peer review only

For peer review only - http://bmjopen.bmj.com/site/about/guidelines.xhtml

ABSTRACT

Introduction

Care of children with cancer is highly specialised and requires well educated, trained and dedicated nurses to provide high quality care. In low and middle income countries (LMICs), the survival rate of children with cancer is low compared to that of high income countries (HICs) due to the limited number of specialised oncology health care professionals especially nurses. To address this problem, a number of paediatric oncology education and training programmes have been developed for nurses. The objective of this scoping review is to describe the existing literature focusing on paediatric oncology nursing education and training programs; to map the content, delivery methods, duration and mode of assessment.

Methods

The review will include articles published in English, from 2012 to 2022, that describe a paediatric oncology nursing education programme, from any setting. The review will be conducted in accordance with Joanna Briggs Institute (JBI) methodology for scoping reviews. A systematic search of literature will be performed in CINAHL, Dimensions, Embase, PubMed, and Scopus. A two-stage standardised screening process will be employed to evaluate eligibility of the articles. All abstracts that will be considered relevant will be reviewed in full text form by the two reviewers independently. Conflicts will be resolved by consensus of all reviewers through a meeting. Data will be extracted from articles included in the scoping review by two independent reviewers using a developed data extraction tool. The results will be reported in extraction tables and diagrams with a narrative summary.

Ethics and Dissemination

This scoping review is part of the multiphase study which obtained ethical clearance from Malawi and Human Research Ethics Committee of the University of the Witwatersrand. The scoping review will be published in a peer reviewed journal. The findings will also be presented at national and international conferences.

For peer review only - http://bmjopen.bmj.com/site/about/guidelines.xhtml

Strengths and limitations of the study

- The proposed scoping review will be conducted in accordance with JBI methodology for scoping reviews.
- A comprehensive systematic search strategy will be conducted. Articles published in English within a ten-year period, that describe a paediatric oncology nursing education and training program, from any setting or geographical location will be included in this review.
- This review will not report on the quality of included studies, but rather identify existing literature for paediatric oncology nursing education and training program.
- The review will map content, delivery methods, duration and mode of assessment for the paediatric oncology nursing education and training programmes.
- Patients and the public were not involved in the design of this scoping review protocol.

INTRODUCTION

Childhood cancer also known as paediatric oncology is a leading cause of deaths among non-communicable diseases in children worldwide [1,2] . Each year, more than 400 000 children are diagnosed with cancer and 80% of these are from low and middle income countries (LMICs) [1,3]. In high income countries (HICs), the survival rate of children with all types of cancer is more than 80% while in LMICs, the survival rate ranges from 5 to 60% [4,5]. The few children that survive in LMICs live with a disability as a result of suboptimal management of the cancer [6]. Late presentation with advanced cancer disease, coupled with other factors such as abandonment of treatment, limited resources for specific cancer treatment, limited neurosurgical options, lack of radiotherapy and intense supportive care have been reported to contribute to poor survival rate of children with cancer in LMICs [7–13].

The ability to respond to the cancer burden has also been affected by the limited number of specialised oncology health care professionals such as nurses in LMICs [14,15]. Nurses form the largest group of health care professionals globally and are

For peer review only - http://bmjopen.bmj.com/site/about/guidelines.xhtml

considered the back bone of the health care system in the management of children with cancer [2]. They provide physical, psychosocial, pharmacological and other supportive care whose aim is to promote quality of life of children with cancer and their survival [16]. As such, nurses working in paediatric oncology settings need to be well prepared to perform their roles with confidence for successful team coordination and positive patient outcomes. However, the lack of nurses with paediatric oncology education and skills has been identified as a major challenge to the implementation of evidence-based oncology nursing care [17].

While acknowledging that specialty education and training is the most ideal to improve outcomes of children with cancer in LMICs, this may not be realised soon in many of these settings because the processes of developing and implementing a curriculum take a long time [18,19]. Consequently, children are at risk of dying of cancer as they continue to get substandard nursing care, in addition to not receiving appropriate anticancer treatment. To address this challenge, nurses working in oncology settings in some LMIC's are offered orientation education and training through a twinning programme [20]. A twinning programme is an approach where a HIC facility partners with a LMIC facility [6,11,20]. A collaborating facility from a HIC provides oncology orientation training to a partner institution in LMIC for a specific period [11,20]. However, few institutions in LMICs benefit from twinning programmes and there is no sustainability when the nurse educator returns to their home country [21]. Other factors that affect effectiveness of the twining programmes include lack of consideration of contextual issues during the training, lack of commitment from the LMIC partner institution and the high cost associated with the twinning program [14]. To address these challenges, Sullivan *et al.*, [22] recommend that hospitals from LMIC's should invest in capacity building of nurses through low-cost education programs relevant to their context to improve patient outcomes.

Care of children with cancer is highly specialised and requires well educated, trained and dedicated nurses to provide high quality care [23]. In most LMICs, the undergraduate curriculum for nurses has limited content for oncology nursing, and as such nurses receive little or no paediatric oncology-specific education during pre-service education [24]. Unlike HICs and some LMICs, where nurses are given an orientation education programme when newly hired or allocated to paediatric oncology settings, many LMICs, do not have such education and training opportunities [15,24].

For peer review only - http://bmjopen.bmj.com/site/about/guidelines.xhtml

Nurses learn to deal with complexities of cancer management on the job through experience, intuition, doctors' instructions and everyone teaches them as they see fit [25,26]. This has also been seen to promote transfer of negative traditions and practices at the work place as nurses may be learning from mistakes [26,27].

Several paediatric oncology education programmes have been developed both in HICs and LMICs. These programmes have helped to prepare nurses with appropriate knowledge, skills and attitudes to become competent clinical nurses in paediatric oncology settings thereby filling the gap of specialty education and training. The published educational programmes have used different methods of teaching such as physical classroom teaching, online learning, mentorship approach and clinical teaching [17,24]. However, no scoping review on paediatric oncology nursing education and training has been conducted. The reviews currently available focus on education offered to Multidisciplinary teams [28,29], the use of simulation-based educational strategies to educate nurses and physicians about cancer care [30] and access to and experience of education for children and adolescents with cancer [31]. In addition, a scoping review of published training and education initiatives for health professionals in LMICs by Karim *et al.*, (2021)[28] was performed to understand the strategies used to train the global oncology workforce. In their review, they found that most educational initiatives targeted physicians and focused on continuing medical education. Almost all the initiatives were done under twinning programmes. From this review, one article described the impact of the initiative on patient outcomes and less than half of the initiatives involved e-learning. As such, authors recommended oncology training and education initiatives for non-physicians in LMICs.

A preliminary search for existing reviews on this topic was conducted in Joanna Briggs Institute, PubMed and CINAHL databases and google scholar. No protocols for a similar review were found. The current scoping review aims at describing the current literature focusing on paediatric oncology nursing education and training programs; to map the content, delivery methods, duration and mode of assessment.

Scoping review questions

The current scoping review aims at answering the following research questions:

For peer review only

For peer review only - http://bmjopen.bmj.com/site/about/guidelines.xhtml

1. What is the content of the paediatric oncology education and training programmes?
2. What delivery methods did the paediatric oncology education and training programmes use?
3. What was the duration of education and training programmes?
4. How were the paediatric oncology education and training programmes assessed?

Inclusion criteria

This review will use the following inclusion criteria using the Participant Context Concept (PCC) mnemonic:

Participant

This review will focus on articles about paediatric oncology nursing education and training programmes. Information for paediatric oncology nursing only will be extracted from literature that combine education and training programmes for adult and paediatric oncology nursing. For studies that focused on multidisciplinary teams, we will extract information for nurses only; and studies from HICs and LMICs will be included.

Concept

The review will include articles that describe a paediatric oncology nursing education and training programme. The programme can be continued education, orientation training or short learning programme. Short learning program in this review is defined as any education and training program offered for a period of 30 minutes to six months. Information to be extracted will include content, delivery methods, duration and mode of assessment.

Context

The review will include articles published in peer reviewed scientific journals and grey literature from the ProQuest Dissertation & Theses Global, paediatric oncology reports and guidelines. Articles written in English language from 2012 to 2022 will be included in the review. A 10-year period is broad enough to provide recent evidence in paediatric oncology nursing. Articles could also be from any setting, or geographical location.

For peer review only

Exclusion criteria

The current review will exclude articles that report on education and training programmes for multidisciplinary teams but do not separate results for nurses and other cadres of health care professionals. This will be difficult to isolate data for nurses only. Articles that report on paediatric oncology nursing education and training programmes as part of a curriculum for a nursing training institution will also be excluded.

METHODOLOGY

The proposed scoping review will be conducted in accordance with JBI methodology for scoping reviews. The following steps will be followed:

- Searching strategy
- Study selection
- Data extraction
- Data analysis and presentation

Step 1: Searching strategy

A systematic search of literature using Medical Subject Headings (MeSH), search terms or phrases, synonyms and alternative terms will be performed. The following search terms /phrases and their synonyms will be used: Paediatric oncology nursing, childhood cancer nursing, continuing education, orientation training, educational programmes, in-service training. Search strings will be created by combining the Boolean operators OR and AND (Appendix 1 search strategy example) The search will be conducted in the following databases: CINAHL, Dimensions, Embase, PubMed, Scopus. The search will aim to identify published articles from peer reviewed journals. The search will include additional sources from the ProQuest Dissertation and Theses Global, paediatric oncology reports and guidelines. Reference lists of relevant articles will be screened to identify additional articles. An experienced librarian in systematic search of literature will adapt the attached structured search strategy (Appendix 1) to all databases depending on their design.

For peer review only - http://bmjopen.bmj.com/site/about/guidelines.xhtml

Step 2: Study selection

Bibliographic results of the searches from all the databases including reference lists will be downloaded and uploaded into Zotero Reference Management Software for deduplication, then uploaded into Joanna Briggs Institute System for the Unified Management, Assessment, and Review of Information (JBI SUMARI) for title and abstract screening. JBI SUMARI is a web-based review application that supports the steps involved in the review process from drafting a protocol, selection of studies, critical appraisal, data extraction and synthesis and also allows one to manage review teams and contributors to the review [32].

A two-stage standardised screening process will be employed to evaluate eligibility of the articles. Two independent reviewers (MDM, FC) will first screen the title and abstracts of the captured articles. Full text articles of the included studies will then be downloaded. Disagreements between the two reviewers will be discussed with the other three reviewers (AS, LM, IK) and final decision will be made. The results of the search will be reported in full in the final scoping review and presented in a Preferred Reporting Items for Systematic Reviews and Meta-Analyses extension for Scoping Reviews (PRISMA-ScR) flow diagram (Figure 1) [33].

Step 3: Data extraction

Two independent reviewers will extract data from included articles. This will be performed using a data extraction tool developed by MDM. Disagreements between the two reviewers will be discussed with the other three reviewers to reach a consensus. The data to be extracted will include: author, year, journal, country, host site/ institution, collaborations, location of training, project title, theory content, practical areas, programme delivery methods, programme duration and mode of assessment.

A draft data extraction tool is provided (see Appendix 2). The draft data extraction tool will be modified and revised as necessary during the data extraction process. Modifications will be detailed in the full scoping review. Any disagreements that arise between the reviewers will be resolved through discussion or with a third reviewer. Assessment of quality of included articles will not be performed as it is not within the scope of this review.

For peer review only - http://bmjopen.bmj.com/site/about/guidelines.xhtml

Step 4: Data analysis and presentation

Content analysis using basic coding of data will be performed. The results will be described narratively, and presented using summary of findings table and flow-diagrams.

Ethics and dissemination

This scoping review is part of the multiphase study. The study obtained ethical clearance from Malawi, the study site (Approval number P.12/21/3537) and Human Research Ethics Committee of the University of the Witwatersrand (M220429). Findings from the scoping review will guide the third phase of the study and will contribute towards a Doctor of Philosophy in Nursing for MDM. The scoping review will be written according to PRISMA-ScR guidelines [33] and published in a peer reviewed journal. The findings will also be presented at national and international conferences.

Protocol registration

The protocol was registered with the Open Science Framework with registration link/number Osf.io/x3q4h

Patient and public Involvement

This study protocol will analyse data from published research and grey literature. Patients or members of the public will not be involved. Patients and/or the public were not involved in the design, or conduct, or reporting, or dissemination plans of this research.

Data availability

Data will be available on request with the permission of the University.

Discussion

To the best of our knowledge, this will be the first scoping review that will map available evidence on paediatric oncology nursing education and training programmes. We

For peer review only - http://bmjopen.bmj.com/site/about/guidelines.xhtml

expect that the results of this review will be of interest to nurses practicing in different paediatric oncology settings, nursing education institutions and nursing regulatory bodies in LMICs as well as HICs.

Acknowledgements

All authors for their contributions.

Competing interests

None declared.

Patient consent for publication

Not applicable.

Author affiliations

[1]School of Nursing, Kamuzu University of Health Sciences, Malawi.

[2]Consortium for Advanced Research Training in Africa, Nairobi, Kenya.

[3] Department of Nursing Education, School of Therapeutic Sciences, Faculty of Health Sciences, University of the Witwatersrand. South Africa.

[4]Library Department, Kamuzu University of Health Sciences, Malawi.

[5]School of Public Health, Faculty of Health Sciences, University of the Witwatersrand. South Africa.

Authors' contributions

The review was drafted and designed by MDM. The revision was performed by FC, AS, IK, JEM. All authors approved the final scoping review protocol and agreed on the submission for publication in a peer reviewed scientific journal.

Funding

This work is supported by the Consortium for Advanced Research Training in Africa (CARTA). CARTA is jointly led by the African Population and Health Research Center and the University of the Witwatersrand and funded by the Carnegie Corporation of New York (Grant No. G-19-57145), Sida (Grant No:**54100113**), Uppsala Monitoring Center, Norwegian Agency for Development Cooperation (Norad), and by the Wellcome Trust [reference no. 107768/Z/15/Z] and the UK Foreign, Commonwealth & Development Office, with support from the Developing Excellence in Leadership,

For peer review only - http://bmjopen.bmj.com/site/about/guidelines.xhtml

Training and Science in Africa (DELTAS Africa) programme. The statements made and views expressed are solely the responsibility of the Fellow. For the purpose of open access, the author has applied a CC BY public copyright license to any Author Accepted Manuscript version arising from this submission."

ORCID iDs

Maureen Majamanda: https://orcid.org/0000-0001-8886-3158

Apatsa Selemani: https://orcid.org/0000-0003-1216-6158

Felix Chisoni: https://orcid.org/0000-003-1798-6446

Irene Kearns: https://orcid.org/0000-0003-2667-3353

Johanna E Maree: https://orcid.org/0000-0002-3153-3040

For peer review only

Reference

1. World Health Organization. Noncommunicable disease facility-based monitoring guidance: framework, indicators and application [Internet]. Geneva: World Health Organization; 2022 [cited 2022 Nov 21]. Available from: https://apps.who.int/iris/handle/10665/364379

2. Pergert P, Sullivan CE, Adde M, Afungchwi GM, Downing J, Hollis R, et al. An ethical imperative: Safety and specialization as nursing priorities of WHO Global Initiative for Childhood Cancer. Pediatric Blood & Cancer [Internet]. 2020 [cited 2022 Oct 19];67(4):e28143. Available from: https://onlinelibrary.wiley.com/doi/abs/10.1002/pbc.28143

3. Lam CG, Howard SC, Bouffet E, Pritchard-Jones K. Science and health for all children with cancer. Science [Internet]. 2019 Mar 15 [cited 2022 Oct 19];363(6432):1182–6. Available from: http://0-search.ebscohost.com.innopac.wits.ac.za/login.aspx?direct=true&AuthType=ip&db=asn&AN=135342497&site=ehost-live&scope=site

4. Denburg A, Arora B, Arora RS, Auste C, Bagai P, Barr R, et al. Access to essential medicines for children with cancer: a joint SIOP-CCI position statement. The Lancet Oncology [Internet]. 2017 Jan [cited 2022 Oct 19];18(1):20–2. Available from: https://linkinghub.elsevier.com/retrieve/pii/S1470204516306520

5. Rodriguez-Galindo C, Friedrich P, Alcasabas P, Antillon F, Banavali S, Castillo L, et al. Toward the Cure of All Children With Cancer Through Collaborative Efforts: Pediatric Oncology As a Global Challenge. J Clin Oncol [Internet]. 2015 Sep 20 [cited 2022 Oct 19];33(27):3065–73. Available from: https://www.ncbi.nlm.nih.gov/pmc/articles/PMC4979198/

6. Hopkins J, Burns E, Eden T. International twinning partnerships: An effective method of improving diagnosis, treatment and care for children with cancer in low-middle income countries. Journal of Cancer Policy [Internet]. 2013 Mar [cited 2022 Oct 19];1(1–2):e8–19. Available from: https://linkinghub.elsevier.com/retrieve/pii/S2213538313000040

7. Chakumatha E, Weijers J, Banda K, Bailey S, Molyneux E, Chagaluka G, et al. Outcome at the end of treatment of patients with common and curable childhood cancer types in Blantyre, Malawi. Pediatr Blood Cancer [Internet]. 2020 Jul [cited 2022 Oct 19];67(7). Available from: https://onlinelibrary.wiley.com/doi/10.1002/pbc.28322

8. Boyle P, Ngoma T, Sullivan R, Brawley O. Cancer in Africa: the way forward. Ecancermedicalscience [Internet]. 2019 Jul 25 [cited 2022 Nov 11];13:953. Available from: https://www.ncbi.nlm.nih.gov/pmc/articles/PMC6722114/

9. Stulac S, Binagwaho A, Tapela NM, Wagner CM, Muhimpundu MA, Ngabo F, et al. Capacity building for oncology programmes in sub-Saharan Africa: the Rwanda experience. The Lancet Oncology [Internet]. 2015 Aug [cited 2022 Oct 19];16(8):e405–13. Available from: https://linkinghub.elsevier.com/retrieve/pii/S1470204515001618

10. Cimiotti JP, Barton SJ, Gorman KEC, Sloane DM, Aiken LH. Nurse Reports on Resource Adequacy in Hospitals that Care for Acutely Ill Children. J Healthc Qual

For peer review only - http://bmjopen.bmj.com/site/about/guidelines.xhtml

[Internet]. 2014 Mar [cited 2022 Oct 19];36(2):25–32. Available from: https://www.ncbi.nlm.nih.gov/pmc/articles/PMC3687020/

11. Day SW, Segovia L, Viveros P, Alqudimat MR, Rivera GK. The Latin American Center for Pediatric Oncology Nursing Education: Development, Implementation, and Accomplishments. Cancer Nursing [Internet]. 2013 Sep [cited 2022 Oct 19];36(5):340–5. Available from: https://journals.lww.com/00002820-201309000-00002

12. Israels T, Borgstein E, Pidini D, Chagaluka G, de Kraker J, Kamiza S, et al. Management of Children With a Wilms Tumor in Malawi, Sub-Saharan Africa. Journal of Pediatric Hematology/Oncology [Internet]. 2012 Nov [cited 2022 Oct 19];34(8):606–10. Available from: https://journals.lww.com/00043426-201211000-00004

13. Moreira C, Nachef MN, Ziamati S, Ladjaj Y, Barsaoui S, Mallon B, et al. Treatment of nephroblastoma in Africa: Results of the first French African pediatric oncology group (GFAOP) study. Pediatric Blood & Cancer [Internet]. 2012 [cited 2022 Oct 19];58(1):37–42. Available from: https://onlinelibrary.wiley.com/doi/abs/10.1002/pbc.23284

14. So WKW, Cummings GG, de Calvo LEA, Day SW, Houlahan K, Nevidjon BM, et al. Enhancement of oncology nursing education in low- and middle-income countries: Challenges and strategies. Journal of Cancer Policy [Internet]. 2016 Jun [cited 2022 Nov 11];8:10–6. Available from: https://linkinghub.elsevier.com/retrieve/pii/S221353831630008X

15. Oti NOO, de Villiers M, Adejumo P, Okumu R, Maliti B, Elkateb N, et al. Strengthening of oncology nursing education and training in Africa in the year of the nurse and midwife: addressing the challenges to improve cancer control in Africa. Ecancermedicalscience [Internet]. 2021 Mar 23 [cited 2022 Oct 19];15:1209. Available from: https://www.ncbi.nlm.nih.gov/pmc/articles/PMC8057781/

16. Challinor JM, Hollis R, Freidank C, Verhoeven C. Educational Needs and Strategies of Pediatric Oncology Nurses in Low- and Middle-Income Countries: An International Society of Pediatric Oncology Pediatric Oncology in Developing Countries Nursing Working Group Initiative. Cancer Nursing [Internet]. 2014 Jul [cited 2022 Oct 19];37(4):E36–47. Available from: https://journals.lww.com/00002820-201407000-00013

17. Day SW, Garcia J, Antillon F, Wilimas JA, McKeon LM, Carty RM, et al. A Sustainable Model for Pediatric Oncology Nursing Education in Low-Income Countries. Pediatr Blood Cancer [Internet]. 2012 Feb [cited 2022 Oct 25];58(2):163–6. Available from: https://www.ncbi.nlm.nih.gov/pmc/articles/PMC4676073/

18. Mailloux CG. Using The Essentials of Baccalaureate Education for Professional Nursing Practice (2008) as a Framework for Curriculum Revision. Journal of Professional Nursing [Internet]. 2011 Nov [cited 2021 Jul 15];27(6):385–9. Available from: https://linkinghub.elsevier.com/retrieve/pii/S8755722311000469

19. Oermann MH. Curriculum Revision: Making Informed Decisions. Nurse Educator [Internet]. 2019 [cited 2021 Jul 15];44(1):1. Available from: http://journals.lww.com/10.1097/NNE.0000000000000630

20. Galassi A, Challinor J. Strengthening the oncology nurse workforce in low-income and middle-income countries. Lancet Oncology [Internet]. 2015 Aug [cited 2022 Nov

For peer review only - http://bmjopen.bmj.com/site/about/guidelines.xhtml

23];16(8):887–8. Available from: https://www.proquest.com/docview/1702711806/abstract/C8B2B9281D954432PQ/1

21. Mitema A, Maree L, Young A. Cancer treatment in Africa: the importance of the role of nursing. Ecancermedicalscience [Internet]. 2019 Jul 25 [cited 2022 Nov 11];13:944. Available from: https://www.ncbi.nlm.nih.gov/pmc/articles/PMC6722106/

22. Sullivan CE, Morrissey L, Day SW, Chen Y, Shirey M, Landier W. Predictors of Hospitals' Nonachievement of Baseline Nursing Standards for Pediatric Oncology. Cancer Nursing [Internet]. 2020 Aug [cited 2022 Nov 11];43(4):E197. Available from: https://journals.lww.com/cancernursingonline/Fulltext/2020/07000/Predictors_of_Hospitals__Nonachievement_of.13.aspx

23. Andersson N, Klang B, Petersson G. Differences in clinical reasoning among nurses working in highly specialised paediatric care. Journal of Clinical Nursing [Internet]. 2012 [cited 2022 Oct 19];21(5–6):870–9. Available from: http://0.onlinelibrary.wiley.com/doi/abs/10.1111/j.1365-2702.2011.03935.x

24. Hockenberry M, Mulemba T, Nedege A, Madumetse K, Higgins J. Distance-Based Education for Nurses Caring for Children With Cancer in Sub-Saharan Africa. J Pediatr Oncol Nurs [Internet]. 2020 Sep [cited 2021 Jul 8];37(5):321–9. Available from: http://journals.sagepub.com/doi/10.1177/1043454220938355

25. Adejumo PO, Akinyemi KF, Anarado AN, Ndikom CM, Ohaeri BM, Okedare OO, et al. State of oncology nursing training and practice in a southwestern Nigerian teaching hospital. Journal of Nursing Education and Practice [Internet]. 2021 May 27 [cited 2022 Nov 20];11(9):68. Available from: https://www.sciedupress.com/journal/index.php/jnep/article/view/20389

26. Tynjälä P. Perspectives into learning at the workplace. Educational Research Review [Internet]. 2008 Jan [cited 2022 Nov 11];3(2):130–54. Available from: https://linkinghub.elsevier.com/retrieve/pii/S1747938X0800002X

27. Hussein R, Everett B, Ramjan LM, Hu W, Salamonson Y. New graduate nurses experiences in a clinical specialty: a follow up study of newcomer perceptions of transitional support. BMC Nursing [Internet]. 2017 [cited 2022 Nov 11];16. Available from: https://www.proquest.com/docview/1925453576/abstract/3249B0D6557E4369PQ/1

28. Karim S, Sunderji Z, Jalink M, Mohamed S, Mallick I, Msadabwe-Chikuni SC, et al. Oncology training and education initiatives in low and middle income countries: a scoping review. Ecancermedicalscience [Internet]. 2021 Sep 30 [cited 2022 Apr 29];15:1296. Available from: https://www.ncbi.nlm.nih.gov/pmc/articles/PMC8580602/

29. Topperzer MK, Hoffmann M, Roug LI, Larsen HB, Lausen B, Schmiegelow K, et al. Unmet need for interprofessional education in paediatric cancer: a scoping review. Support Care Cancer [Internet]. 2019 [cited 2022 Nov 23];27(10):3627–37. Available from: https://www.ncbi.nlm.nih.gov/pmc/articles/PMC6726701/

30. Silva A, Galica J, Woo K, Ross-White A, Luctkar-Flude M. The use of simulation-based education in cancer care: a scoping review protocol. International Journal of Healthcare Simulation [Internet]. 00:00:00.0 [cited 2022 Nov 11];1(2):37–41. Available from: https://www.ijohs.com/article/doi/10.54531/dlvs9567

For peer review only - http://bmjopen.bmj.com/site/about/guidelines.xhtml

31. Bryan G, Kelly P, Chesters H, Franklin J, Griffiths H, Langton L, et al. Access to and experience of education for children and adolescents with cancer: a scoping review protocol. Syst Rev [Internet]. 2021 Dec [cited 2022 Apr 29];10(1):167. Available from: https://systematicreviewsjournal.biomedcentral.com/articles/10.1186/s13643-021-01723-4

32. Piper C. System for the Unified Management, Assessment, and Review of Information (SUMARI). J Med Libr Assoc [Internet]. 2019 Oct [cited 2022 Nov 20];107(4):634–6. Available from: https://www.ncbi.nlm.nih.gov/pmc/articles/PMC6774554/

33. Tricco AC, Lillie E, Zarin W, O'Brien KK, Colquhoun H, Levac D, et al. PRISMA Extension for Scoping Reviews (PRISMA-ScR): Checklist and Explanation. Ann Intern Med [Internet]. 2018 Oct 2 [cited 2022 Feb 21];169(7):467–73. Available from: https://www.acpjournals.org/doi/10.7326/M18-0850

34. Hussein R, Everett B, Ramjan LM, Hu W, Salamonson Y. New graduate nurses' experiences in a clinical specialty: a follow up study of newcomer perceptions of transitional support. BMC Nurs [Internet]. 2017 Jul 28 [cited 2022 Oct 19];16:42. Available from: https://www.ncbi.nlm.nih.gov/pmc/articles/PMC5534089/

For peer review only

For peer review only - http://bmjopen.bmj.com/site/about/guidelines.xhtml

Figure 1: PRISMA Flow Chart of study identification & selection

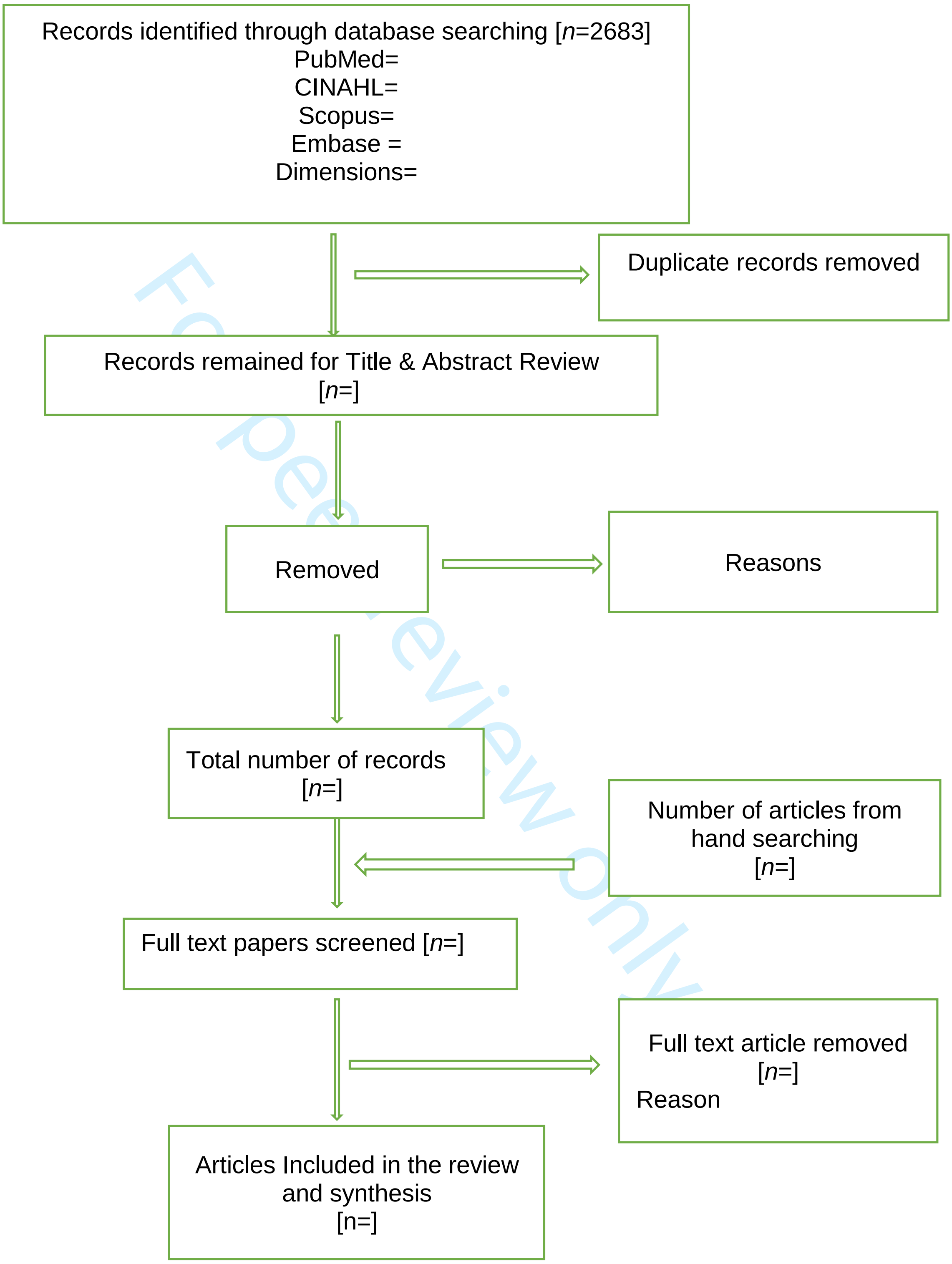

For peer review only

Appendix 1: Scoping Review Search strategy example PubMed 17/07/2022

History and Search Details

Download Delete

Search	Actions	Details	Query	Results	Time
#5	•••	>	Search: **#1 AND #2 AND #3** Filters: **from 2012 - 2022**	45	18:22:53
#4	•••	>	Search: **#1 AND #2 AND #3**	84	18:22:22
#3	•••	>	Search: **(Paediatric oncology[MeSH Terms]) OR (Paediatric oncology nursing[Text Word])**	110,129	18:19:56
#2	•••	>	Search: **((((continuing education[Text Word]) OR (Education, Continuing[MeSH Terms])) OR (In service education[Text Word])) OR (In service training[Text Word])) OR (continuous professional development[Text Word])**	70,694	18:17:14
#1	•••	>	Search: **(((Childhood cancer[Text Word]) OR (Paediatric cancer[Text Word])) OR (neoplasms[MeSH Terms])) OR (neoplasms[Text Word])**	3,738,096	18:10:33

Showing 1 to 5 of 5 entries

peer review only

Appendix 2: A draft data extraction tool

	Studies			
**Item**	**1**	**2**	**3**	**4 etc.**
Author				
Year				
Journal				
Country				
Host site/ institution				
Collaborations				
Partner country				
Partner institution				
Location of training				
Project title				
Target population				
Theory content				
Practical areas				
Programme delivery methods				
Programme duration				
Mode of assessment				

For peer review only

Preferred Reporting Items for Systematic reviews and Meta-Analyses extension for Scoping Reviews (PRISMA-ScR) Checklist

SECTION	ITEM	PRISMA-ScR CHECKLIST ITEM	REPORTED ON PAGE #
**TITLE**			
Title	1	Identify the report as a scoping review.	
**ABSTRACT**			
Structured summary	2	Provide a structured summary that includes (as applicable): background, objectives, eligibility criteria, sources of evidence, charting methods, results, and conclusions that relate to the review questions and objectives.	
**INTRODUCTION**			
Rationale	3	Describe the rationale for the review in the context of what is already known. Explain why the review questions/objectives lend themselves to a scoping review approach.	
Objectives	4	Provide an explicit statement of the questions and objectives being addressed with reference to their key elements (e.g., population or participants, concepts, and context) or other relevant key elements used to conceptualize the review questions and/or objectives.	
**METHODS**			
Protocol and registration	5	Indicate whether a review protocol exists; state if and where it can be accessed (e.g., a Web address); and if available, provide registration information, including the registration number.	
Eligibility criteria	6	Specify characteristics of the sources of evidence used as eligibility criteria (e.g., years considered, language, and publication status), and provide a rationale.	
Information sources*	7	Describe all information sources in the search (e.g., databases with dates of coverage and contact with authors to identify additional sources), as well as the date the most recent search was executed.	
Search	8	Present the full electronic search strategy for at least 1 database, including any limits used, such that it could be repeated.	
Selection of sources of evidence†	9	State the process for selecting sources of evidence (i.e., screening and eligibility) included in the scoping review.	
Data charting process‡	10	Describe the methods of charting data from the included sources of evidence (e.g., calibrated forms or forms that have been tested by the team before their use, and whether data charting was done independently or in duplicate) and any processes for obtaining and confirming data from investigators.	
Data items	11	List and define all variables for which data were sought and any assumptions and simplifications made.	
Critical appraisal of individual sources of evidence§	12	If done, provide a rationale for conducting a critical appraisal of included sources of evidence; describe the methods used and how this information was used in any data synthesis (if appropriate).	
Synthesis of results	13	Describe the methods of handling and summarizing the data that were charted.	

For peer review only

SECTION	ITEM	PRISMA-ScR CHECKLIST ITEM	REPORTED ON PAGE #
**RESULTS**			
Selection of sources of evidence	14	Give numbers of sources of evidence screened, assessed for eligibility, and included in the review, with reasons for exclusions at each stage, ideally using a flow diagram.	
Characteristics of sources of evidence	15	For each source of evidence, present characteristics for which data were charted and provide the citations.	
Critical appraisal within sources of evidence	16	If done, present data on critical appraisal of included sources of evidence (see item 12).	
Results of individual sources of evidence	17	For each included source of evidence, present the relevant data that were charted that relate to the review questions and objectives.	
Synthesis of results	18	Summarize and/or present the charting results as they relate to the review questions and objectives.	
**DISCUSSION**			
Summary of evidence	19	Summarize the main results (including an overview of concepts, themes, and types of evidence available), link to the review questions and objectives, and consider the relevance to key groups.	
Limitations	20	Discuss the limitations of the scoping review process.	
Conclusions	21	Provide a general interpretation of the results with respect to the review questions and objectives, as well as potential implications and/or next steps.	
**FUNDING**			
Funding	22	Describe sources of funding for the included sources of evidence, as well as sources of funding for the scoping review. Describe the role of the funders of the scoping review.	

JBI = Joanna Briggs Institute; PRISMA-ScR = Preferred Reporting Items for Systematic reviews and Meta-Analyses extension for Scoping Reviews.

* Where *sources of evidence* (see second footnote) are compiled from, such as bibliographic databases, social media platforms, and Web sites.

† A more inclusive/heterogeneous term used to account for the different types of evidence or data sources (e.g., quantitative and/or qualitative research, expert opinion, and policy documents) that may be eligible in a scoping review as opposed to only studies. This is not to be confused with *information sources* (see first footnote).

‡ The frameworks by Arksey and O'Malley (6) and Levac and colleagues (7) and the JBI guidance (4, 5) refer to the process of data extraction in a scoping review as data charting.

§ The process of systematically examining research evidence to assess its validity, results, and relevance before using it to inform a decision. This term is used for items 12 and 19 instead of "risk of bias" (which is more applicable to systematic reviews of interventions) to include and acknowledge the various sources of evidence that may be used in a scoping review (e.g., quantitative and/or qualitative research, expert opinion, and policy document).

From: Tricco AC, Lillie E, Zarin W, O'Brien KK, Colquhoun H, Levac D, et al. PRISMA Extension for Scoping Reviews (PRISMAScR): Checklist and Explanation. Ann Intern Med. 2018;169:467–473. doi: 10.7326/M18-0850.

For peer review only - http://bmjopen.bmj.com/site/about/guidelines.xhtml

BMJ Open

Paediatric oncology nursing education and training programmes: A scoping review protocol

Journal:	*BMJ Open*
Manuscript ID	bmjopen-2022-070694.R1
Article Type:	Protocol
Date Submitted by the Author:	28-Apr-2023

Complete List of Authors:	Majamanda, Maureen; Kamuzu University of Health Sciences, Child Health Nursing<br>Chisoni, Felix; Kamuzu University of Health Sciences, Library<br>Selemani, Apatsa; Kamuzu University of Health Sciences, Library<br>Kearns, Irene; University of the Witwatersrand Johannesburg Faculty of Health Sciences, Nursing Education<br>Maree, Johanna; University of the Witwatersrand Johannesburg, Nursing Education

**Primary Subject Heading**:	Oncology
Secondary Subject Heading:	Paediatrics
Keywords:	ONCOLOGY, Paediatric oncology < ONCOLOGY, PUBLIC HEALTH, Nursing Care

SCHOLARONE™
Manuscripts

Title:

Paediatric oncology nursing education and training programmes: A scoping review protocol

AUTHORS:

Maureen D. Majamanda[1,2,3], Felix Chisoni[4], Apatsa Selemani [2,4,5], Irene Kearns[3], Johanna E. Maree[3]

Corresponding author

Maureen D. Majamanda, Kamuzu University of Health Sciences, School of Nursing, Chipatala Avenue, P.O Box 415, Blantyre, Malawi.

Email: mdmajamanda@kuhes.ac.mw

Cell phone: +265 (0) 992 160 415

Fax: +(265) 018753941

Co-authors

Felix Chisoni, Kamuzu University of Health Sciences, Blantyre, Malawi.

Apatsa Seleani, Kamuzu University of Health Sciences, Blantyre, Malawi.

Irene Kearns, University of the Witwatersrand, Johannesburg, South Africa.

Johanna E. Maree, University of the Witwatersrand, Johannesburg, South Africa.

Key words

Paediatric oncology nursing, continuing education, orientation training, educational programmes, in-service training

Word count

2603

For peer review only

ABSTRACT

Introduction

Care of children with cancer is highly specialised and requires well educated, trained and dedicated nurses to provide high quality care. In low and middle income countries (LMICs), the survival rate of children with cancer is low compared to that of high income countries (HICs) due to the limited number of specialised oncology health care professionals' especially nurses. To address this problem, a number of paediatric oncology education and training programmes have been developed for nurses. The objective of this scoping review is to describe the existing literature focusing on paediatric oncology nursing education and training programmes; to map the content, delivery methods, duration and mode of assessment.

Methods

The review will include articles published in English, from 2012 to 2022, that describe a paediatric oncology nursing education programme, from any setting. The review will be conducted in accordance with Joanna Briggs Institute (JBI) methodology for scoping reviews. A systematic search of literature will be performed in CINAHL, Dimensions, Embase, PubMed, and Scopus. A two-stage standardised screening process will be employed to evaluate eligibility of the articles. All abstracts that will be considered relevant will be reviewed in full text form by the two reviewers independently. Conflicts will be resolved by consensus of all reviewers through a meeting. Data will be extracted from articles included in the scoping review by two independent reviewers using a developed data extraction tool. The results will be reported in extraction tables and diagrams with a narrative summary.

Ethics and Dissemination

This scoping review is part of the multiphase study which obtained ethical clearance from Malawi and Human Research Ethics Committee of the University of the Witwatersrand. The scoping review will be published in a peer reviewed journal. The findings will also be presented at national and international conferences.

For peer review only - http://bmjopen.bmj.com/site/about/guidelines.xhtml

Strengths and limitations of the study

- The proposed scoping review will be conducted in accordance with JBI methodology for scoping reviews.
- A comprehensive systematic search strategy will be conducted. Articles published in English within a ten-year period, that describe a paediatric oncology nursing education and training programme, from High, Low and Middle income countries will be included in this review.
- This review will not report on the quality of included studies, but rather identify existing literature for paediatric oncology nursing education and training programme.
- The review will map content, cultural/ contextual issues, delivery methods, duration and mode of assessment for the paediatric oncology nursing education and training programmes.
- Patients and the public were not involved in the design of this scoping review protocol.
- Inclusion of studies published in English language only and searching for articles in few databases will limit the findings thereby providing incomplete mapping of the evidence

INTRODUCTION

Childhood cancer also known as paediatric oncology is a leading cause of deaths among non-communicable diseases in children worldwide [1,2] . Each year, more than 400 000 children are diagnosed with cancer and 80% of these are from low and middle income countries (LMICs) [1,3]. In high income countries (HICs), the survival rate of children with all types of cancer is more than 80% while in LMICs, the survival rate ranges from 5 to 60% [4,5]. The few children that survive in LMICs live with a disability as a result of suboptimal management of the cancer [6]. Late presentation with advanced cancer disease, coupled with other factors such as abandonment of treatment, limited resources for specific cancer treatment, limited neurosurgical options, lack of radiotherapy and intense supportive care have been reported to contribute to poor survival rate of children with cancer in LMICs [7–13].

The ability to respond to the cancer burden has also been affected by the limited number of specialised oncology health care professionals such as nurses in LMICs

For peer review only - http://bmjopen.bmj.com/site/about/guidelines.xhtml

[14,15]. Nurses form the largest group of health care professionals globally and are considered the back bone of the health care system in the management of children with cancer [2]. They provide physical, psychosocial, pharmacological and other supportive care whose aim is to promote quality of life of children with cancer and their survival [16]. As such, nurses working in paediatric oncology settings need to be well prepared to perform their roles with confidence for successful team coordination and positive patient outcomes. However, the lack of nurses with paediatric oncology education and skills has been identified as a major challenge to the implementation of evidence-based oncology nursing care [17].

Care of children with cancer is highly specialised and requires well educated, trained and dedicated nurses to provide high quality care [18]. In most LMICs the undergraduate curriculum for nurses has limited content for oncology nursing, and as such nurses receive little or no paediatric oncology-specific education during pre-service education [19]. Unlike HICs and some LMICs, where nurses are given an orientation education programme when newly hired or allocated to paediatric oncology settings, many LMICs, do not have such education and training opportunities [15,19]. Nurses learn to deal with complexities of cancer management on the job through experience, intuition and doctors' instructions [20,21]. With lack of speciality training, nurses are not able to effectively manage children with cancer and this negatively affects the outcomes of care.

While acknowledging that specialty education and training is the most ideal way to improve outcomes of children with cancer in LMICs, this may not be realised soon in many of these settings because the processes of developing and implementing a curriculum take a long time [22,23]. Consequently, children are at risk of dying of cancer as they continue to be managed by untrained nurses who are not able to fully meet the demands of pediatric cancer care due to lack of extensive knowledge and skills such as psychosocial support[24]. To address this challenge, nurses working in oncology settings in some LMIC's are offered orientation education and training through a twinning programme [25]. A twinning programme is an approach where a HIC facility partners with a LMIC facility [6,11,25]. A collaborating facility from a HIC provides oncology orientation training to a partner institution in LMIC for a specific period [11,25]. However, few institutions in LMICs benefit from twinning programmes and there is no sustainability when the nurse educator returns to their home country [26]. Other factors

For peer review only - http://bmjopen.bmj.com/site/about/guidelines.xhtml

that affect effectiveness of the twinning programmes include lack of consideration of contextual issues during the training, lack of commitment from the LMIC partner institution and the high cost associated with the twinning programme [14]. To address these challenges, Sullivan *et al.*, [27] recommend that hospitals from LMIC's should invest in capacity building of nurses through low-cost education programmes relevant to their context to improve patient outcomes.

Several paediatric oncology education programmes have been developed both in HICs and LMICs. These programmes have helped to prepare nurses with appropriate knowledge, skills and attitudes to become competent clinical nurses in paediatric oncology settings thereby filling the gap of specialty education and training. The published educational programmes have used different methods of teaching such as physical classroom teaching, online learning, mentorship approach and clinical teaching [17,19]. However, no scoping review on paediatric oncology nursing education and training has been conducted. The reviews currently available focus on education offered to Multidisciplinary teams [28,29], the use of simulation-based educational strategies to educate nurses and physicians about cancer care [30] and access to and experience of education for children and adolescents with cancer [31]. In addition, a scoping review of published training and education initiatives for health professionals in LMICs by [28] was performed to understand the strategies used to train the global oncology workforce. In their review, they found that most educational initiatives targeted physicians and focused on continuing medical education. Almost all the initiatives were done under twinning programmes. From this review, one article described the impact of the initiative on patient outcomes and less than half of the initiatives involved e-learning. As such, authors recommended oncology training and education initiatives for non-physicians in LMICs.

A preliminary search for existing reviews on this topic was conducted in Joanna Briggs Institute, PubMed and CINAHL databases and Google Scholar. No protocols for a similar review were found. The current scoping review aims at describing the current literature focusing on paediatric oncology nursing education and training programmes; to map the content, delivery methods, duration and mode of assessment.

For peer review only - http://bmjopen.bmj.com/site/about/guidelines.xhtml

Scoping review questions

The current scoping review aims at answering the following research questions:

1. What is the content of the paediatric oncology nursing education and training programmes?
2. What delivery methods did the paediatric oncology nursing education and training programmes use?
3. What was the duration of the paediatric oncology nursing education and training programmes?
4. How were the paediatric oncology nursing education and training programmes assessed?

Inclusion criteria

This review will use the following inclusion criteria using the Participant Context Concept (PCC) mnemonic:

Participant

This review will focus on articles about paediatric oncology nursing education and training programmes. Information for paediatric oncology nursing only will be extracted from literature that combine education and training programmes for adult and paediatric oncology nursing. For studies that focused on multidisciplinary teams, we will extract information for nurses only; and studies from HICs and LMICs will be included.

Concept

The review will include articles that describe a paediatric oncology nursing education and training programme. The programme can be continued education, orientation training or short learning programme. Short learning programme in this review is defined as any education and training programme offered for a period of two hours to six months. Information to be extracted will include content, delivery methods, duration and mode of assessment.

Context

The review will include articles published in peer reviewed scientific journals and grey literature from the ProQuest Dissertation & Theses Global, paediatric oncology reports

For peer review only - http://bmjopen.bmj.com/site/about/guidelines.xhtml

and guidelines. Articles written in English language from 2012 to 2022 will be included in the review. A 10-year period is broad enough to provide recent evidence in paediatric oncology nursing. Articles could also be from LMICs and HICs.

Exclusion criteria

The current review will exclude articles that report on education and training programmes for multidisciplinary teams but do not separate results for nurses and other cadres of health care professionals. This will be difficult to isolate data for nurses only. Articles that report on paediatric oncology nursing education and training programmes as part of a nursing curriculum for pre service training at an academic institution such as a University will also be excluded because the focus for this scoping review is on programmes for qualified nurses.

METHODOLOGY

The proposed scoping review will be conducted in accordance with JBI methodology for scoping reviews [32]. The following steps will be followed:

- Searching strategy
- Study selection
- Data extraction
- Data analysis and presentation

Step 1: Searching strategy

A systematic search of literature using Medical Subject Headings (MeSH), index terms, search terms or phrases, synonyms and alternative terms will be performed. The following search terms /phrases and their synonyms will be used: Paediatric oncology nursing, childhood cancer nursing, continuing education, orientation training, educational programmes, in-service training. Search strings will be created by combining the Boolean operators OR and AND (Appendix 1 search strategy example) The search will be conducted in the following databases: CINAHL, Dimensions, Embase, PubMed, Scopus and Education Resources Information Center (ERIC). The search will aim to identify published articles from peer reviewed journals. The search will include grey literature from the ProQuest Dissertation and Theses Global, paediatric oncology reports and guidelines as well as paediatric oncology national and

For peer review only

For peer review only - http://bmjopen.bmj.com/site/about/guidelines.xhtml

international society websites. Reference lists of relevant articles will be screened to identify additional articles. An experienced librarian in systematic search of literature will adapt the attached structured search strategy (Appendix 1) to all databases depending on their design.

Step 2: Study selection

Bibliographic results of the searches from all the databases including reference lists will be downloaded and uploaded into Zotero Reference Management Software for deduplication, then uploaded into Joanna Briggs Institute System for the Unified Management, Assessment, and Review of Information (JBI SUMARI) for title and abstract screening. JBI SUMARI is a web-based review application that supports the steps involved in the review process from drafting a protocol, selection of studies, critical appraisal, data extraction and synthesis and also allows one to manage review teams and contributors to the review [33].

A two-stage standardised screening process will be employed to evaluate eligibility of the articles. Two independent reviewers (MDM, FC) will first screen the title and abstracts of the captured articles. Full text articles of the included studies will then be downloaded. Disagreements between the two reviewers will be discussed with the other three reviewers (AS, LM, IK) and final decision will be made. The results of the search will be reported in full in the final scoping review and presented in a Preferred Reporting Items for Systematic Reviews and Meta-Analyses extension for Scoping Reviews (PRISMA-ScR) flow diagram (Figure 1) [34].

Step 3: Data extraction

Two independent reviewers will extract data from included articles. This will be performed using a data extraction tool developed by MDM. The data extraction tool will be tested on a sample of papers to determine its practicality prior to the data extraction process. The data to be extracted will include: author, year, journal, country, host site/ institution, collaborations, location of training, project title, theory content, practical areas, cultural/ contextual issues, programme delivery methods, programme duration, mode of assessment and theoretical framework underpinning the training programme.

For peer review only - http://bmjopen.bmj.com/site/about/guidelines.xhtml

A draft data extraction tool is provided (see Appendix 2). The draft data extraction tool will be modified and revised as necessary during the data extraction process. Modifications will be detailed in the full scoping review. Any disagreements that arise between the reviewers will be resolved through discussion or with a third reviewer. Assessment of quality of included articles will not be performed as it is not within the scope of this review.

Step 4: Data analysis and presentation

Content analysis using basic coding of data will be performed. The results will be described narratively, and presented using summary of findings table and flow-diagrams. The evidence will be summarised in relation to the purpose of this review and programmes that are relevant in LMICs will be identified.

Ethics and dissemination

This scoping review is part of the multiphase study. The study obtained ethical clearance from Malawi, the study site (Approval number P.12/21/3537) and Human Research Ethics Committee of the University of the Witwatersrand (M220429). Findings from the scoping review will guide the third phase of the study and will contribute towards a Doctor of Philosophy in Nursing for MDM. The scoping review will be written according to PRISMA-ScR guidelines [34] and published in a peer reviewed journal. The findings will also be presented at national and international conferences.

Protocol registration

The protocol was registered with the Open Science Framework with registration link/number Osf.io/x3q4h

Patient and public Involvement

This study protocol will analyse data from published research and grey literature. Patients or members of the public will not be involved. Patients and/or the public were

For peer review only - http://bmjopen.bmj.com/site/about/guidelines.xhtml

For peer review only

not involved in the design, or conduct, or reporting, or dissemination plans of this research.

Data availability

Data will be available on request with the permission of the University.

Discussion

To the best of our knowledge, this will be the first scoping review that will map available evidence on paediatric oncology nursing education and training programmes. We expect that the results of this review will be of interest to nurses practicing in different paediatric oncology settings, nursing education institutions and nursing regulatory bodies in LMICs as well as HICs. The findings will also inform the development of a paediatric oncology short learning programme for nurses in Malawi and other similar contexts.

Acknowledgements

All authors for their contributions.

Competing interests

None declared.

Patient consent for publication

Not applicable.

Author affiliations

[1]School of Nursing, Kamuzu University of Health Sciences, Malawi.

[2]Consortium for Advanced Research Training in Africa, Nairobi, Kenya.

[3] Department of Nursing Education, School of Therapeutic Sciences, Faculty of Health Sciences, University of the Witwatersrand. South Africa.

[4]Library Department, Kamuzu University of Health Sciences, Malawi.

[5]School of Public Health, Faculty of Health Sciences, University of the Witwatersrand. South Africa.

Authors' contributions

For peer review only - http://bmjopen.bmj.com/site/about/guidelines.xhtml

The review was drafted and designed by MDM. The revision was performed by FC, AS, IK, JEM. All authors approved the final scoping review protocol and agreed on the submission for publication in a peer reviewed scientific journal.

Funding

This work is supported by the Consortium for Advanced Research Training in Africa (CARTA). CARTA is jointly led by the African Population and Health Research Center and the University of the Witwatersrand and funded by the Carnegie Corporation of New York (Grant No. G-19-57145), Sida (Grant No:**54100113**), Uppsala Monitoring Center, Norwegian Agency for Development Cooperation (Norad), and by the Wellcome Trust [reference no. 107768/Z/15/Z] and the UK Foreign, Commonwealth & Development Office, with support from the Developing Excellence in Leadership, Training and Science in Africa (DELTAS Africa) programme. The statements made and views expressed are solely the responsibility of the Fellow. For the purpose of open access, the author has applied a CC BY public copyright license to any Author Accepted Manuscript version arising from this submission."

ORCID iDs

Maureen Majamanda: https://orcid.org/0000-0001-8886-3158

Apatsa Selemani: https://orcid.org/0000-0003-1216-6158

Felix Chisoni: https://orcid.org/0000-003-1798-6446

Irene Kearns: https://orcid.org/0000-0003-2667-3353

Johanna E Maree: https://orcid.org/0000-0002-3153-3040

Reference

1. World Health Organization. Noncommunicable disease facility-based monitoring guidance: framework, indicators and application [Internet]. Geneva: World Health Organization; 2022 [cited 2022 Nov 21]. Available from: https://apps.who.int/iris/handle/10665/364379

2. Pergert P, Sullivan CE, Adde M, Afungchwi GM, Downing J, Hollis R, et al. An ethical imperative: Safety and specialization as nursing priorities of WHO Global Initiative for Childhood Cancer. Pediatric Blood & Cancer [Internet]. 2020 [cited 2022 Oct 19];67(4):e28143. Available from: https://onlinelibrary.wiley.com/doi/abs/10.1002/pbc.28143

3. Lam CG, Howard SC, Bouffet E, Pritchard-Jones K. Science and health for all children with cancer. Science [Internet]. 2019 Mar 15 [cited 2022 Oct 19];363(6432):1182–6. Available from: http://0-search.ebscohost.com.innopac.wits.ac.za/login.aspx?direct=true&AuthType=ip&db=asn&AN=135342497&site=ehost-live&scope=site

4. Denburg A, Arora B, Arora RS, Auste C, Bagai P, Barr R, et al. Access to essential medicines for children with cancer: a joint SIOP-CCI position statement. The Lancet Oncology [Internet]. 2017 Jan [cited 2022 Oct 19];18(1):20–2. Available from: https://linkinghub.elsevier.com/retrieve/pii/S1470204516306520

5. Rodriguez-Galindo C, Friedrich P, Alcasabas P, Antillon F, Banavali S, Castillo L, et al. Toward the Cure of All Children With Cancer Through Collaborative Efforts: Pediatric Oncology As a Global Challenge. J Clin Oncol [Internet]. 2015 Sep 20 [cited 2022 Oct 19];33(27):3065–73. Available from: https://www.ncbi.nlm.nih.gov/pmc/articles/PMC4979198/

6. Hopkins J, Burns E, Eden T. International twinning partnerships: An effective method of improving diagnosis, treatment and care for children with cancer in low-middle income countries. Journal of Cancer Policy [Internet]. 2013 Mar [cited 2022 Oct 19];1(1–2):e8–19. Available from: https://linkinghub.elsevier.com/retrieve/pii/S2213538313000040

7. Chakumatha E, Weijers J, Banda K, Bailey S, Molyneux E, Chagaluka G, et al. Outcome at the end of treatment of patients with common and curable childhood cancer types in Blantyre, Malawi. Pediatr Blood Cancer [Internet]. 2020 Jul [cited 2022 Oct 19];67(7). Available from: https://onlinelibrary.wiley.com/doi/10.1002/pbc.28322

8. Boyle P, Ngoma T, Sullivan R, Brawley O. Cancer in Africa: the way forward. Ecancermedicalscience [Internet]. 2019 Jul 25 [cited 2022 Nov 11];13:953. Available from: https://www.ncbi.nlm.nih.gov/pmc/articles/PMC6722114/

9. Stulac S, Binagwaho A, Tapela NM, Wagner CM, Muhimpundu MA, Ngabo F, et al. Capacity building for oncology programmes in sub-Saharan Africa: the Rwanda experience. The Lancet Oncology [Internet]. 2015 Aug [cited 2022 Oct 19];16(8):e405–13. Available from: https://linkinghub.elsevier.com/retrieve/pii/S1470204515001618

10. Cimiotti JP, Barton SJ, Gorman KEC, Sloane DM, Aiken LH. Nurse Reports on Resource Adequacy in Hospitals that Care for Acutely Ill Children. J Healthc Qual

For peer review only - http://bmjopen.bmj.com/site/about/guidelines.xhtml

[Internet]. 2014 Mar [cited 2022 Oct 19];36(2):25–32. Available from: https://www.ncbi.nlm.nih.gov/pmc/articles/PMC3687020/

11. Day SW, Segovia L, Viveros P, Alqudimat MR, Rivera GK. The Latin American Center for Pediatric Oncology Nursing Education: Development, Implementation, and Accomplishments. Cancer Nursing [Internet]. 2013 Sep [cited 2022 Oct 19];36(5):340–5. Available from: https://journals.lww.com/00002820-201309000-00002

12. Israels T, Borgstein E, Pidini D, Chagaluka G, de Kraker J, Kamiza S, et al. Management of Children With a Wilms Tumor in Malawi, Sub-Saharan Africa. Journal of Pediatric Hematology/Oncology [Internet]. 2012 Nov [cited 2022 Oct 19];34(8):606–10. Available from: https://journals.lww.com/00043426-201211000-00004

13. Moreira C, Nachef MN, Ziamati S, Ladjaj Y, Barsaoui S, Mallon B, et al. Treatment of nephroblastoma in Africa: Results of the first French African pediatric oncology group (GFAOP) study. Pediatric Blood & Cancer [Internet]. 2012 [cited 2022 Oct 19];58(1):37–42. Available from: https://onlinelibrary.wiley.com/doi/abs/10.1002/pbc.23284

14. So WKW, Cummings GG, de Calvo LEA, Day SW, Houlahan K, Nevidjon BM, et al. Enhancement of oncology nursing education in low- and middle-income countries: Challenges and strategies. Journal of Cancer Policy [Internet]. 2016 Jun [cited 2022 Nov 11];8:10–6. Available from: https://linkinghub.elsevier.com/retrieve/pii/S221353831630008X

15. Oti NOO, de Villiers M, Adejumo P, Okumu R, Maliti B, Elkateb N, et al. Strengthening of oncology nursing education and training in Africa in the year of the nurse and midwife: addressing the challenges to improve cancer control in Africa. Ecancermedicalscience [Internet]. 2021 Mar 23 [cited 2022 Oct 19];15:1209. Available from: https://www.ncbi.nlm.nih.gov/pmc/articles/PMC8057781/

16. Challinor JM, Hollis R, Freidank C, Verhoeven C. Educational Needs and Strategies of Pediatric Oncology Nurses in Low- and Middle-Income Countries: An International Society of Pediatric Oncology Pediatric Oncology in Developing Countries Nursing Working Group Initiative. Cancer Nursing [Internet]. 2014 Jul [cited 2022 Oct 19];37(4):E36–47. Available from: https://journals.lww.com/00002820-201407000-00013

17. Day SW, Garcia J, Antillon F, Wilimas JA, McKeon LM, Carty RM, et al. A Sustainable Model for Pediatric Oncology Nursing Education in Low-Income Countries. Pediatr Blood Cancer [Internet]. 2012 Feb [cited 2022 Oct 25];58(2):163–6. Available from: https://www.ncbi.nlm.nih.gov/pmc/articles/PMC4676073/

18. Andersson N, Klang B, Petersson G. Differences in clinical reasoning among nurses working in highly specialised paediatric care. Journal of Clinical Nursing [Internet]. 2012 [cited 2022 Oct 19];21(5–6):870–9. Available from: http://0.onlinelibrary.wiley.com/doi/abs/10.1111/j.1365-2702.2011.03935.x

19. Hockenberry M, Mulemba T, Nedege A, Madumetse K, Higgins J. Distance-Based Education for Nurses Caring for Children With Cancer in Sub-Saharan Africa. J Pediatr Oncol Nurs [Internet]. 2020 Sep [cited 2021 Jul 8];37(5):321–9. Available from: http://journals.sagepub.com/doi/10.1177/1043454220938355

For peer review only - http://bmjopen.bmj.com/site/about/guidelines.xhtml

20. Adejumo PO, Akinyemi KF, Anarado AN, Ndikom CM, Ohaeri BM, Okedare OO, et al. State of oncology nursing training and practice in a southwestern Nigerian teaching hospital. Journal of Nursing Education and Practice [Internet]. 2021 May 27 [cited 2022 Nov 20];11(9):68. Available from: https://www.sciedupress.com/journal/index.php/jnep/article/view/20389

21. Tynjälä P. Perspectives into learning at the workplace. Educational Research Review [Internet]. 2008 Jan [cited 2022 Nov 11];3(2):130–54. Available from: https://linkinghub.elsevier.com/retrieve/pii/S1747938X0800002X

22. Mailloux CG. Using The Essentials of Baccalaureate Education for Professional Nursing Practice (2008) as a Framework for Curriculum Revision. Journal of Professional Nursing [Internet]. 2011 Nov [cited 2021 Jul 15];27(6):385–9. Available from: https://linkinghub.elsevier.com/retrieve/pii/S8755722311000469

23. Oermann MH. Curriculum Revision: Making Informed Decisions. Nurse Educator [Internet]. 2019 [cited 2021 Jul 15];44(1):1. Available from: http://journals.lww.com/10.1097/NNE.0000000000000630

24. Day SW, Garcia J, Antillon F, Wilimas JA, McKeon LM, Carty RM, et al. A sustainable model for pediatric oncology nursing education in low-income countries: Oncology Nursing in Low-Income Countries. Pediatr Blood Cancer [Internet]. 2012 Feb [cited 2023 Feb 6];58(2):163–6. Available from: https://onlinelibrary.wiley.com/doi/10.1002/pbc.24007

25. Galassi A, Challinor J. Strengthening the oncology nurse workforce in low-income and middle-income countries. Lancet Oncology [Internet]. 2015 Aug [cited 2022 Nov 23];16(8):887–8. Available from: https://www.proquest.com/docview/1702711806/abstract/C8B2B9281D954432PQ/1

26. Mitema A, Maree L, Young A. Cancer treatment in Africa: the importance of the role of nursing. Ecancermedicalscience [Internet]. 2019 Jul 25 [cited 2022 Nov 11];13:944. Available from: https://www.ncbi.nlm.nih.gov/pmc/articles/PMC6722106/

27. Sullivan CE, Morrissey L, Day SW, Chen Y, Shirey M, Landier W. Predictors of Hospitals' Nonachievement of Baseline Nursing Standards for Pediatric Oncology. Cancer Nursing [Internet]. 2020 Aug [cited 2022 Nov 11];43(4):E197. Available from: https://journals.lww.com/cancernursingonline/Fulltext/2020/07000/Predictors_of_Hospitals__Nonachievement_of.13.aspx

28. Karim S, Sunderji Z, Jalink M, Mohamed S, Mallick I, Msadabwe-Chikuni SC, et al. Oncology training and education initiatives in low and middle income countries: a scoping review. Ecancermedicalscience [Internet]. 2021 Sep 30 [cited 2022 Apr 29];15:1296. Available from: https://www.ncbi.nlm.nih.gov/pmc/articles/PMC8580602/

29. Topperzer MK, Hoffmann M, Roug LI, Larsen HB, Lausen B, Schmiegelow K, et al. Unmet need for interprofessional education in paediatric cancer: a scoping review. Support Care Cancer [Internet]. 2019 [cited 2022 Nov 23];27(10):3627–37. Available from: https://www.ncbi.nlm.nih.gov/pmc/articles/PMC6726701/

30. Silva A, Galica J, Woo K, Ross-White A, Luctkar-Flude M. The use of simulation-based education in cancer care: a scoping review protocol. International Journal of Healthcare Simulation [Internet]. 00:00:00.0 [cited 2022 Nov 11];1(2):37–41. Available from: https://www.ijohs.com/article/doi/10.54531/dlvs9567

For peer review only - http://bmjopen.bmj.com/site/about/guidelines.xhtml

31. Bryan G, Kelly P, Chesters H, Franklin J, Griffiths H, Langton L, et al. Access to and experience of education for children and adolescents with cancer: a scoping review protocol. Syst Rev [Internet]. 2021 Dec [cited 2022 Apr 29];10(1):167. Available from: https://systematicreviewsjournal.biomedcentral.com/articles/10.1186/s13643-021-01723-4

32. Peters MDJ, Marnie C, Tricco AC, Pollock D, Munn Z, Alexander L, et al. Updated methodological guidance for the conduct of scoping reviews. JBI Evidence Synthesis [Internet]. 2020 Oct [cited 2023 Jan 31];18(10):2119–26. Available from: https://journals.lww.com/10.11124/JBIES-20-00167

33. Piper C. System for the Unified Management, Assessment, and Review of Information (SUMARI). J Med Libr Assoc [Internet]. 2019 Oct [cited 2022 Nov 20];107(4):634–6. Available from: https://www.ncbi.nlm.nih.gov/pmc/articles/PMC6774554/

34. Tricco AC, Lillie E, Zarin W, O'Brien KK, Colquhoun H, Levac D, et al. PRISMA Extension for Scoping Reviews (PRISMA-ScR): Checklist and Explanation. Ann Intern Med [Internet]. 2018 Oct 2 [cited 2022 Feb 21];169(7):467–73. Available from: https://www.acpjournals.org/doi/10.7326/M18-0850

35. Hussein R, Everett B, Ramjan LM, Hu W, Salamonson Y. New graduate nurses' experiences in a clinical specialty: a follow up study of newcomer perceptions of transitional support. BMC Nurs [Internet]. 2017 Jul 28 [cited 2022 Oct 19];16:42. Available from: https://www.ncbi.nlm.nih.gov/pmc/articles/PMC5534089/

Figure legend:

Figure-1-prisma-flow-diagram

For peer review only

For peer review only - http://bmjopen.bmj.com/site/about/guidelines.xhtml

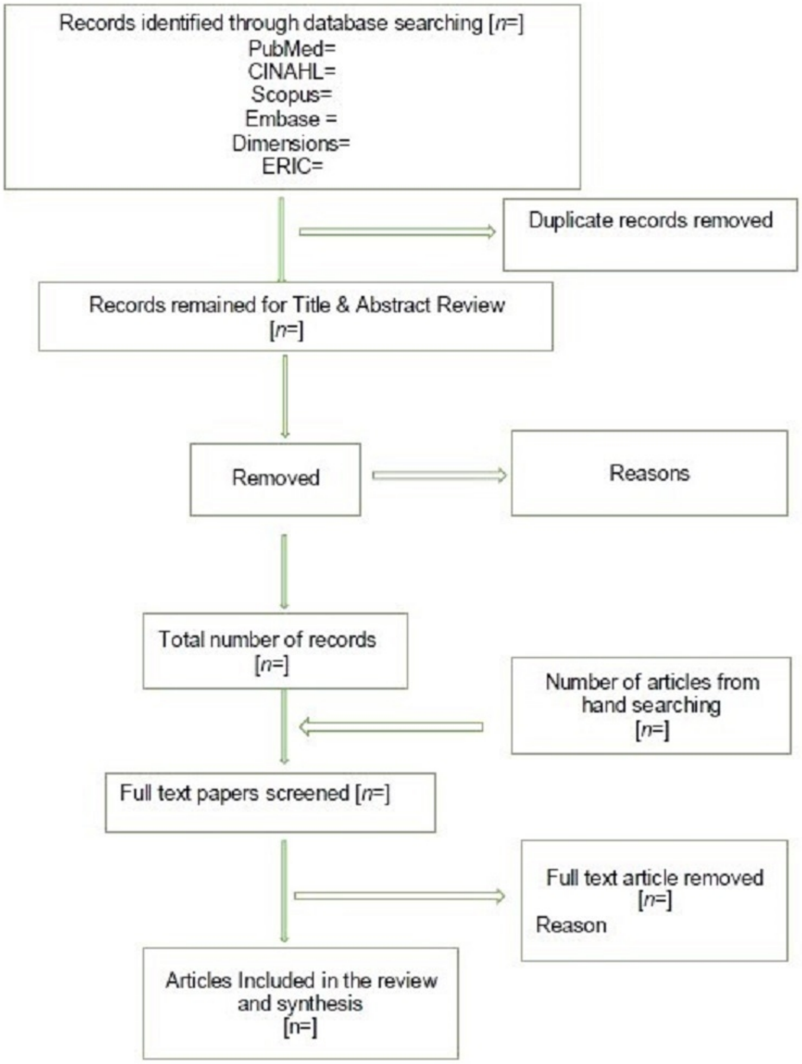

Figure 1: PRISMA Flow Chart of study identification & selection

Figure-1-prisma-flow-diagram

70x93mm (300 x 300 DPI)

Appendix 1: Database: PUBMED

Set #		Results
1	((((neoplasm*[MeSH Terms])) OR (paediatric cancer [Title/Abstract])) OR (childhood cancer [Text Word])) OR (neoplasm*[Text Word]) Filters: in the last 10 years	1,458,541
2	((((continuing education [Text Word]) OR (Education, continuing [MeSH Terms])) OR (In service education[Text Word])) OR (Inservice training[Text Word])) OR (Continuous Professional development[Text Word]) Filters: in the last 10 years	18,517
3	(Paediatric oncology [MeSH Terms]) OR (Paediatric oncology nursing [Text Word]) Filters: in the last 10 years	66,410
4	((((((neoplasm*[MeSH Terms])) OR (paediatric cancer[Title/Abstract])) OR (childhood cancer[Text Word])) OR (neoplasm*[Text Word]) AND (y_10[Filter])) AND (((((continuing education[Text Word]) OR (Education, continuing[MeSH Terms])) OR (In service education[Text Word])) OR (Inservice training[Text Word])) OR (Continuous Professional development[Text Word]) AND (y_10[Filter]))) AND ((Paediatric oncology[MeSH Terms]) OR (Paediatric oncology nursing[Text Word]) AND (y_10[Filter])) Filters: in the last 10 years	45

For peer review only

Appendix 2: A draft data extraction tool

	Studies			
**Item**	**1**	**2**	**3**	**4 etc.**
Author				
Year				
Journal				
Country				
Host site/ institution				
Collaborations				
Partner country				
Partner institution				
Location of training				
Project title				
Target population				
Theory content				
Practical areas				
Programme delivery methods				
Programme duration				
Mode of assessment				
Cultural/ contextual issues				
Theoretical framework				

For peer review only

Preferred Reporting Items for Systematic reviews and Meta-Analyses extension for Scoping Reviews (PRISMA-ScR) Checklist

SECTION	ITEM	PRISMA-ScR CHECKLIST ITEM	REPORTED ON PAGE #
**TITLE**			
Title	1	Identify the report as a scoping review.	
**ABSTRACT**			
Structured summary	2	Provide a structured summary that includes (as applicable): background, objectives, eligibility criteria, sources of evidence, charting methods, results, and conclusions that relate to the review questions and objectives.	
**INTRODUCTION**			
Rationale	3	Describe the rationale for the review in the context of what is already known. Explain why the review questions/objectives lend themselves to a scoping review approach.	
Objectives	4	Provide an explicit statement of the questions and objectives being addressed with reference to their key elements (e.g., population or participants, concepts, and context) or other relevant key elements used to conceptualize the review questions and/or objectives.	
**METHODS**			
Protocol and registration	5	Indicate whether a review protocol exists; state if and where it can be accessed (e.g., a Web address); and if available, provide registration information, including the registration number.	
Eligibility criteria	6	Specify characteristics of the sources of evidence used as eligibility criteria (e.g., years considered, language, and publication status), and provide a rationale.	
Information sources*	7	Describe all information sources in the search (e.g., databases with dates of coverage and contact with authors to identify additional sources), as well as the date the most recent search was executed.	
Search	8	Present the full electronic search strategy for at least 1 database, including any limits used, such that it could be repeated.	
Selection of sources of evidence†	9	State the process for selecting sources of evidence (i.e., screening and eligibility) included in the scoping review.	
Data charting process‡	10	Describe the methods of charting data from the included sources of evidence (e.g., calibrated forms or forms that have been tested by the team before their use, and whether data charting was done independently or in duplicate) and any processes for obtaining and confirming data from investigators.	
Data items	11	List and define all variables for which data were sought and any assumptions and simplifications made.	
Critical appraisal of individual sources of evidence§	12	If done, provide a rationale for conducting a critical appraisal of included sources of evidence; describe the methods used and how this information was used in any data synthesis (if appropriate).	
Synthesis of results	13	Describe the methods of handling and summarizing the data that were charted.	

For peer review only

SECTION	ITEM	PRISMA-ScR CHECKLIST ITEM	REPORTED ON PAGE #
**RESULTS**			
Selection of sources of evidence	14	Give numbers of sources of evidence screened, assessed for eligibility, and included in the review, with reasons for exclusions at each stage, ideally using a flow diagram.	
Characteristics of sources of evidence	15	For each source of evidence, present characteristics for which data were charted and provide the citations.	
Critical appraisal within sources of evidence	16	If done, present data on critical appraisal of included sources of evidence (see item 12).	
Results of individual sources of evidence	17	For each included source of evidence, present the relevant data that were charted that relate to the review questions and objectives.	
Synthesis of results	18	Summarize and/or present the charting results as they relate to the review questions and objectives.	
**DISCUSSION**			
Summary of evidence	19	Summarize the main results (including an overview of concepts, themes, and types of evidence available), link to the review questions and objectives, and consider the relevance to key groups.	
Limitations	20	Discuss the limitations of the scoping review process.	
Conclusions	21	Provide a general interpretation of the results with respect to the review questions and objectives, as well as potential implications and/or next steps.	
**FUNDING**			
Funding	22	Describe sources of funding for the included sources of evidence, as well as sources of funding for the scoping review. Describe the role of the funders of the scoping review.	

JBI = Joanna Briggs Institute; PRISMA-ScR = Preferred Reporting Items for Systematic reviews and Meta-Analyses extension for Scoping Reviews.
* Where *sources of evidence* (see second footnote) are compiled from, such as bibliographic databases, social media platforms, and Web sites.
† A more inclusive/heterogeneous term used to account for the different types of evidence or data sources (e.g., quantitative and/or qualitative research, expert opinion, and policy documents) that may be eligible in a scoping review as opposed to only studies. This is not to be confused with *information sources* (see first footnote).
‡ The frameworks by Arksey and O'Malley (6) and Levac and colleagues (7) and the JBI guidance (4, 5) refer to the process of data extraction in a scoping review as data charting.
§ The process of systematically examining research evidence to assess its validity, results, and relevance before using it to inform a decision. This term is used for items 12 and 19 instead of "risk of bias" (which is more applicable to systematic reviews of interventions) to include and acknowledge the various sources of evidence that may be used in a scoping review (e.g., quantitative and/or qualitative research, expert opinion, and policy document).

From: Tricco AC, Lillie E, Zarin W, O'Brien KK, Colquhoun H, Levac D, et al. PRISMA Extension for Scoping Reviews (PRISMAScR): Checklist and Explanation. Ann Intern Med. 2018;169:467–473. doi: 10.7326/M18-0850.

For peer review only - http://bmjopen.bmj.com/site/about/guidelines.xhtml

BMJ Open

Paediatric oncology nursing education and training programmes: A scoping review protocol

Journal:	*BMJ Open*
Manuscript ID	bmjopen-2022-070694.R2
Article Type:	Protocol
Date Submitted by the Author:	14-Sep-2023
Complete List of Authors:	Majamanda, Maureen; Kamuzu University of Health Sciences, Child Health Nursing<br>Chisoni, Felix; Kamuzu University of Health Sciences, Library<br>Selemani, Apatsa; Kamuzu University of Health Sciences, Library<br>Kearns, Irene; University of the Witwatersrand Johannesburg Faculty of Health Sciences, Nursing Education<br>Maree, Johanna; University of the Witwatersrand Johannesburg, Nursing Education
**Primary Subject Heading**:	Oncology
Secondary Subject Heading:	Paediatrics
Keywords:	ONCOLOGY, Paediatric oncology < ONCOLOGY, PUBLIC HEALTH, Nursing Care

SCHOLARONE™
Manuscripts

Title:

Paediatric oncology nursing education and training programmes: A scoping review protocol

AUTHORS:

Maureen D. Majamanda[1,2,3], Felix Chisoni[4], Apatsa Selemani [2,4,5], Irene Kearns[3], Johanna E. Maree[3]

Corresponding author

Maureen D. Majamanda, Kamuzu University of Health Sciences, School of Nursing, Chipatala Avenue, P.O Box 415, Blantyre, Malawi.

Email: mdmajamanda@kuhes.ac.mw

Cell phone: +265 (0) 992 160 415

Fax: +(265) 018753941

Co-authors

Felix Chisoni, Kamuzu University of Health Sciences, Blantyre, Malawi.

Apatsa Selemani, Kamuzu University of Health Sciences, Blantyre, Malawi.

Irene Kearns, University of the Witwatersrand, Johannesburg, South Africa.

Johanna E. Maree, University of the Witwatersrand, Johannesburg, South Africa.

Key words

Paediatric oncology nursing, continuing education, orientation training, educational programmes, in-service training

Word count

2792

For peer review only - http://bmjopen.bmj.com/site/about/guidelines.xhtml

ABSTRACT

Introduction

The care of children with cancer is a highly specialised field which requires well educated, trained and dedicated nurses to provide high quality care. In low- and middle-income countries (LMICs), the survival rate of children with cancer is low as compared to that of high-income countries (HICs) due to the limited number of specialised oncology health care professionals, especially nurses. To address this problem, a number of paediatric oncology education and training programmes have been developed for nurses. The objective of this scoping review is to describe the existing literature focusing on paediatric oncology nursing education and training programmes; to map the content, delivery methods, duration and mode of assessment.

Methods

The review will include articles published in English, from 2012 to 2022, that describe a paediatric oncology nursing education programme, from any setting. The review will follow Joanna Briggs Institute (JBI) methodology for scoping reviews guidelines. A systematic search of literature will be performed in CINAHL, Dimensions, Embase, PubMed, and Scopus. A two-stage standardised screening process will be employed to evaluate eligibility of the articles. All abstracts that will be considered relevant will be reviewed in full text form by the two reviewers independently. Conflicts will be resolved by consensus of all reviewers through a meeting. Data will be extracted by two independent reviewers using a developed data extraction tool. The results will be reported in extraction tables and diagrams with a narrative summary.

Ethics and Dissemination

This scoping review is part of the multiphase study which obtained ethical clearance from College of Medicine Research Ethics committee (COMREC) in Malawi and Human Research Ethics Committee (HREC) of the University of Witwatersrand, South Africa. The scoping review will be published in a peer reviewed journal. The findings will also be presented at national and international conferences.

Strengths and limitations of the study

- The proposed scoping review will be conducted in accordance with JBI methodology for scoping reviews.

For peer review only

- A comprehensive systematic search strategy will be conducted. Articles to be included will be those published in English within a ten-year period, that describe a paediatric oncology nursing education and training programme, from High, Low- and Middle-income countries. This review will not report on the quality of included studies, but rather identify existing literature for paediatric oncology nursing education and training programme.
- The review will map content, cultural/ contextual issues, delivery methods, duration and mode of assessment for the paediatric oncology nursing education and training programmes.

- Patients and the public were not involved in the design of this scoping review protocol.
- The inclusion of studies published in English language only and systematic search of a few selected databases will limit the findings, thereby providing incomplete mapping of evidence.

INTRODUCTION

Childhood cancer, also known as paediatric oncology, is a leading cause of death among children with non-communicable diseases worldwide. (1,2) Each year, more than 400 000 children are diagnosed with cancer and 80% of these are from low and middle income countries (LMICs). (1,3) In high income countries (HICs), the survival rate of children with all types of cancer is more than 80% while in LMICs, the survival rate ranges from 5 to 60%. (4,5) The few children that survive in LMICs live with a disability as a result of suboptimal management of the cancer. (6) Late presentation with advanced cancer disease, coupled with other factors such as abandonment of treatment, limited resources for specific cancer treatment, limited neurosurgical options, lack of radiotherapy and intense supportive, care have been reported to contribute to poor survival rate of children with cancer in LMICs. (7–13)

The ability to respond to the cancer burden in LMICs has also been affected by the limited number of specialised oncology health care professionals, such as nurses. (14,15) Nurses form the largest group of health care professionals globally and are considered the back bone of the health care system in the management of children with cancer. (2) They provide physical, psychosocial, pharmacological and other supportive care aimed at promoting quality of life of children with cancer and their survival. (16) As such, nurses working in paediatric oncology settings need to be well

For peer review only - http://bmjopen.bmj.com/site/about/guidelines.xhtml

For peer review only

prepared to perform their roles with confidence for successful team coordination and positive patient outcomes. However, the lack of nurses with paediatric oncology education and skills has been identified as a major challenge to the implementation of evidence-informed oncology nursing care. (17)

Care of children with cancer is highly specialised and requires well educated, trained and dedicated nurses to provide high quality care. (18) In most LMICs the undergraduate curriculum for nurses has limited content for oncology nursing, and as such nurses receive little or no paediatric oncology-specific education during pre-service education. (19) Unlike HICs and some LMICs, where nurses are given an orientation education programme when newly hired or allocated to paediatric oncology settings, many LMICs, do not have such education and training opportunities. (15,19) Nurses learn to deal with complexities of cancer management on the job through experience, intuition and doctors' instructions. (20,21) With lack of speciality training, nurses may not be familiar with aspects of care specific to cancer patients and this may negatively affect the outcomes of care. (22)

While acknowledging that specialty education and training is the most ideal way to improve outcomes of children with cancer in LMICs, this may not be realised soon in many of these settings because the processes of developing and implementing a curriculum take a long time. (23,24) Consequently, health outcomes of children with cancer will continue to be poor as nurses managing them are not able to fully meet the specific and complex demands of pediatric cancer care due to lack of extensive knowledge and skills such as psychosocial support and safe administration of chemotherapy. (25) To address this challenge, nurses working in oncology settings in some LMIC's are offered orientation education and training through a twinning programme. (26) A twinning programme is an approach where a HIC facility partners with a LMIC facility. (6,11,26) A collaborating facility from a HIC provides oncology orientation training to a partner institution in LMIC for a specific period. (11,26) However, few institutions in LMICs benefit from twinning programmes and there is no sustainability when the nurse educator returns to their home country. (27) Other factors that affect effectiveness of the twinning programmes include lack of consideration of contextual issues during the training, lack of commitment from the LMIC partner institution and the high cost associated with the twinning programme. (14) To address these challenges, Sullivan *et al.*, (28) recommend that hospitals

For peer review only - http://bmjopen.bmj.com/site/about/guidelines.xhtml

from LMIC's should invest in capacity building of nurses through low-cost education programmes relevant to their context to improve patient outcomes.

Several paediatric oncology education programmes have been developed both in HICs and LMICs. These programmes have helped to prepare nurses with appropriate knowledge, skills and attitudes to become competent clinical nurses in paediatric oncology settings, thereby filling the gap of specialty education and training. The published educational programmes have used different methods of teaching such as physical classroom teaching, online learning, mentorship approach and clinical teaching. (17,19) However, no scoping review on paediatric oncology nursing education and training has been conducted. The reviews currently available focus on education offered to multidisciplinary teams, (29,30) the use of simulation-based educational strategies to educate nurses and physicians about cancer care, (31) and access to and experience of education for children and adolescents with cancer. (32) In addition, a scoping review of published training and education initiatives for health professionals in LMICs by Karim et al.,(29) was performed to understand the strategies used to train the global oncology workforce. In their review, they found that most educational initiatives targeted physicians and focused on continuing medical education. Almost all the initiatives were done under twinning programmes. From this review, one article described the impact of the initiative on patient outcomes and less than half of the initiatives involved e-learning. As such, authors recommended oncology training and education initiatives for non-physicians in LMICs.

A preliminary search for existing reviews on this topic was conducted in PubMed and Scopus databases. No protocols for a similar review were found. The current scoping review aims at describing the current literature focusing on paediatric oncology nursing education and training programmes; to map the content, delivery methods, duration and mode of assessment.

Scoping review questions

The current scoping review aims at answering the following research questions:

1. What is the content of the paediatric oncology nursing education and training programmes?
2. What delivery methods did the paediatric oncology nursing education and training programmes use?

For peer review only - http://bmjopen.bmj.com/site/about/guidelines.xhtml

3. What was the duration of the paediatric oncology nursing education and training programmes?
4. How were the paediatric oncology nursing education and training programmes assessed?

Inclusion criteria

This review will use the following inclusion criteria using the Participant, Context, and Concept (PCC) mnemonic:

Participant

This review will focus on articles about paediatric oncology nursing education and training programmes. Information on paediatric oncology nursing only will be extracted from literature that combine education and training programmes for adult and paediatric oncology nursing. For studies that focused on multidisciplinary teams, we will extract information for nurses only.

Concept

The review will include articles that describe a paediatric oncology nursing education and training programme. The programme can be continued education, orientation training or short learning programme. Short learning programme in this review is defined as any education and training programme offered for a period of two hours to six months. This definition has been developed by the reviewers based on preliminary search and own experience with short learning programmes. Information to be extracted will include content, delivery methods, duration and mode of assessment.

Context

The review will include articles published in peer reviewed scientific journals and grey literature from the ProQuest Dissertation & Theses Global, paediatric oncology reports and guidelines. Articles written in English language from 2012 to 2022 will be included in the review. A 10-year period is broad enough to provide recent evidence in paediatric oncology nursing. Articles from both LMICs and HICs will be included.

Exclusion criteria

The current review will exclude articles that report on education and training programmes for multidisciplinary teams but do not separate results for nurses and

For peer review only - http://bmjopen.bmj.com/site/about/guidelines.xhtml

other cadres of health care professionals as it will be difficult to isolate data for nurses only. Articles that report on paediatric oncology nursing education and training programmes as part of a nursing curriculum for pre-service training at an academic institution such as a University will also be excluded because the focus for this scoping review is on programmes for qualified nurses.

METHODOLOGY

The proposed scoping review will be conducted from February to September, 2023 and in accordance with the JBI methodology for scoping reviews. (33) The following steps will be followed:

- Searching strategy
- Study selection
- Data extraction
- Data analysis and presentation

Step 1: Searching strategy

A systematic search of literature using Medical Subject Headings (MeSH), index terms, search terms or phrases, synonyms and alternative terms will be performed. The following search terms /phrases and their synonyms will be used: paediatric oncology nursing, childhood cancer nursing, continuing education, orientation training, educational programmes, in-service training. Search strings will be created by combining the Boolean operators OR and AND (see Appendix 1 preliminary search strategy) The search will be conducted in the following databases: CINAHL, Dimensions, Embase, PubMed, Scopus and Education Resources Information Center (ERIC). The search will aim to identify published articles from peer reviewed journals. The search will include grey literature from the ProQuest Dissertation and Theses Global, paediatric oncology reports and guidelines as well as paediatric oncology national and international society websites. Reference lists of relevant articles will be screened to identify additional articles. An experienced librarian in systematic search of literature will adapt the attached structured search strategy to all databases depending on their design (Appendix 1).

Step 2: Study selection

Bibliographic results of the searches from all the databases including reference lists will be downloaded and uploaded into Zotero Reference Management Software for

For peer review only - http://bmjopen.bmj.com/site/about/guidelines.xhtml

deduplication, then uploaded into Joanna Briggs Institute System for the Unified Management, Assessment, and Review of Information (JBI SUMARI) for title and abstract screening. JBI SUMARI is a web-based review application that supports the steps involved in the review process from drafting a protocol, selection of studies, critical appraisal, data extraction and synthesis. It also allows one to manage review teams and contributors to the review. (34)

A two-stage standardised screening process will be employed to evaluate eligibility of the articles. Two independent reviewers (MDM, FC) will first screen the title and abstracts of the captured articles. Full text articles of the included studies will then be downloaded. Disagreements between the two reviewers will be discussed with the other three reviewers (AS, LM, IK) and final decision will be made. The results of the search will be reported in full in the final scoping review.

Step 3: Data extraction

Two independent reviewers will extract data from included articles. This will be performed using a data extraction tool developed by MDM. The data extraction tool will be tested on a sample of papers to determine its practicality prior to the data extraction process. The data to be extracted will include: author, year, journal, country, host site/ institution, collaborations, location of training, project title, theory content, practical areas, cultural/ contextual issues, programme delivery methods, programme duration, mode of assessment and theoretical framework underpinning the training programme.

A draft data extraction tool is provided (see Appendix 2). The draft data extraction tool will be modified and revised as necessary during the data extraction process. Modifications will be detailed in the full scoping review. Any disagreements that arise between the reviewers will be resolved through discussion or with a third reviewer. Assessment of quality of included articles will not be performed as it is not within the scope of this review.

Step 4: Data analysis and presentation

Content analysis using basic coding of data will be performed. The results will be described narratively, and presented using summary of findings table and flow-

For peer review only - http://bmjopen.bmj.com/site/about/guidelines.xhtml

diagrams. The evidence will be summarised in relation to the purpose of this review and programmes that are relevant in LMICs will be identified.

Ethics and dissemination

This scoping review is part of the multiphase study. The study obtained ethical clearance from Malawi, the study site (Approval number P.12/21/3537) and Human Research Ethics Committee of the University of the Witwatersrand (M220429). Findings from the scoping review will guide the third phase of the study and will contribute towards a Doctor of Philosophy in Nursing for MDM. The scoping review will be published in a peer reviewed journal. The findings will also be presented at national and international conferences.

Protocol registration

The protocol was registered with the Open Science Framework with registration link/number Osf.io/x3q4h

Patient and public Involvement

None

Data availability

Data will be available on request with the permission of the University.

Discussion

To the best of our knowledge, this will be the first scoping review that will map available evidence on paediatric oncology nursing education and training programmes. We expect that the results of this review will be of interest to nurses practicing in different paediatric oncology settings, nursing education institutions and nursing regulatory bodies in LMICs as well as HICs. The findings will also inform the development of a paediatric oncology short learning programme for nurses in Malawi and other similar contexts.

Acknowledgements

All authors for their contributions.

For peer review only

Competing interests

None declared.

Patient consent for publication

Not applicable.

Author affiliations

[1]School of Nursing, Kamuzu University of Health Sciences, Malawi.
[2]Consortium for Advanced Research Training in Africa, Nairobi, Kenya.
[3] Department of Nursing Education, School of Therapeutic Sciences, Faculty of Health Sciences, University of the Witwatersrand. South Africa.
[4]Library Department, Kamuzu University of Health Sciences, Malawi.
[5]School of Public Health, Faculty of Health Sciences, University of the Witwatersrand. South Africa.

Authors' contributions

The review was drafted and designed by MDM. The revision was performed by FC, AS, IK, JEM. All authors approved the final scoping review protocol and agreed on the submission for publication in a peer reviewed scientific journal.

Funding

This work is supported by the Consortium for Advanced Research Training in Africa (CARTA). CARTA is jointly led by the African Population and Health Research Center and the University of the Witwatersrand and funded by the Carnegie Corporation of New York (Grant No. G-19-57145), Sida (Grant No:**54100113**), Uppsala Monitoring Center, Norwegian Agency for Development Cooperation (Norad), and by the Wellcome Trust [reference no. 107768/Z/15/Z] and the UK Foreign, Commonwealth & Development Office, with support from the Developing Excellence in Leadership, Training and Science in Africa (DELTAS Africa) programme. The statements made and views expressed are solely the responsibility of the Fellow. For the purpose of open access, the author has applied a CC BY public copyright license to any Author Accepted Manuscript version arising from this submission."

ORCID iDs

Maureen Majamanda: https://orcid.org/0000-0001-8886-3158

For peer review only - http://bmjopen.bmj.com/site/about/guidelines.xhtml

Apatsa Selemani: https://orcid.org/0000-0003-1216-6158

Felix Chisoni: https://orcid.org/0000-003-1798-6446

Irene Kearns: https://orcid.org/0000-0003-2667-3353

Johanna E Maree: https://orcid.org/0000-0002-3153-3040

For peer review only

For peer review only - http://bmjopen.bmj.com/site/about/guidelines.xhtml

Reference

1. World Health Organization. Noncommunicable disease facility-based monitoring guidance: framework, indicators and application [Internet]. Geneva: World Health Organization; 2022 [cited 2022 Nov 21]. Available from: https://apps.who.int/iris/handle/10665/364379

2. Pergert P, Sullivan CE, Adde M, Afungchwi GM, Downing J, Hollis R, et al. An ethical imperative: Safety and specialization as nursing priorities of WHO Global Initiative for Childhood Cancer. Pediatric Blood & Cancer [Internet]. 2020 [cited 2022 Oct 19];67(4):e28143. Available from: https://onlinelibrary.wiley.com/doi/abs/10.1002/pbc.28143

3. Lam CG, Howard SC, Bouffet E, Pritchard-Jones K. Science and health for all children with cancer. Science [Internet]. 2019 Mar 15 [cited 2022 Oct 19];363(6432):1182–6. Available from: http://0-search.ebscohost.com.innopac.wits.ac.za/login.aspx?direct=true&AuthType=ip&db=asn&AN=135342497&site=ehost-live&scope=site

4. Denburg A, Arora B, Arora RS, Auste C, Bagai P, Barr R, et al. Access to essential medicines for children with cancer: a joint SIOP-CCI position statement. The Lancet Oncology [Internet]. 2017 Jan [cited 2022 Oct 19];18(1):20–2. Available from: https://linkinghub.elsevier.com/retrieve/pii/S1470204516306520

5. Rodriguez-Galindo C, Friedrich P, Alcasabas P, Antillon F, Banavali S, Castillo L, et al. Toward the Cure of All Children With Cancer Through Collaborative Efforts: Pediatric Oncology As a Global Challenge. J Clin Oncol [Internet]. 2015 Sep 20 [cited 2022 Oct 19];33(27):3065–73. Available from: https://www.ncbi.nlm.nih.gov/pmc/articles/PMC4979198/

6. Hopkins J, Burns E, Eden T. International twinning partnerships: An effective method of improving diagnosis, treatment and care for children with cancer in low-middle income countries. Journal of Cancer Policy [Internet]. 2013 Mar [cited 2022 Oct 19];1(1–2):e8–19. Available from: https://linkinghub.elsevier.com/retrieve/pii/S2213538313000040

7. Chakumatha E, Weijers J, Banda K, Bailey S, Molyneux E, Chagaluka G, et al. Outcome at the end of treatment of patients with common and curable childhood cancer types in Blantyre, Malawi. Pediatr Blood Cancer [Internet]. 2020 Jul [cited 2022 Oct 19];67(7). Available from: https://onlinelibrary.wiley.com/doi/10.1002/pbc.28322

8. Boyle P, Ngoma T, Sullivan R, Brawley O. Cancer in Africa: the way forward. Ecancermedicalscience [Internet]. 2019 Jul 25 [cited 2022 Nov 11];13:953. Available from: https://www.ncbi.nlm.nih.gov/pmc/articles/PMC6722114/

9. Stulac S, Binagwaho A, Tapela NM, Wagner CM, Muhimpundu MA, Ngabo F, et al. Capacity building for oncology programmes in sub-Saharan Africa: the Rwanda experience. The Lancet Oncology [Internet]. 2015 Aug [cited 2022 Oct 19];16(8):e405–13. Available from: https://linkinghub.elsevier.com/retrieve/pii/S1470204515001618

10. Cimiotti JP, Barton SJ, Gorman KEC, Sloane DM, Aiken LH. Nurse Reports on Resource Adequacy in Hospitals that Care for Acutely Ill Children. J Healthc Qual [Internet]. 2014 Mar [cited 2022 Oct 19];36(2):25–32. Available from: https://www.ncbi.nlm.nih.gov/pmc/articles/PMC3687020/

For peer review only - http://bmjopen.bmj.com/site/about/guidelines.xhtml

11. Day SW, Segovia L, Viveros P, Alqudimat MR, Rivera GK. The Latin American Center for Pediatric Oncology Nursing Education: Development, Implementation, and Accomplishments. Cancer Nursing [Internet]. 2013 Sep [cited 2022 Oct 19];36(5):340–5. Available from: https://journals.lww.com/00002820-201309000-00002

12. Israels T, Borgstein E, Pidini D, Chagaluka G, de Kraker J, Kamiza S, et al. Management of Children With a Wilms Tumor in Malawi, Sub-Saharan Africa. Journal of Pediatric Hematology/Oncology [Internet]. 2012 Nov [cited 2022 Oct 19];34(8):606–10. Available from: https://journals.lww.com/00043426-201211000-00004

13. Moreira C, Nachef MN, Ziamati S, Ladjaj Y, Barsaoui S, Mallon B, et al. Treatment of nephroblastoma in Africa: Results of the first French African pediatric oncology group (GFAOP) study. Pediatric Blood & Cancer [Internet]. 2012 [cited 2022 Oct 19];58(1):37–42. Available from: https://onlinelibrary.wiley.com/doi/abs/10.1002/pbc.23284

14. So WKW, Cummings GG, de Calvo LEA, Day SW, Houlahan K, Nevidjon BM, et al. Enhancement of oncology nursing education in low- and middle-income countries: Challenges and strategies. Journal of Cancer Policy [Internet]. 2016 Jun [cited 2022 Nov 11];8:10–6. Available from: https://linkinghub.elsevier.com/retrieve/pii/S221353831630008X

15. Oti NOO, de Villiers M, Adejumo P, Okumu R, Maliti B, Elkateb N, et al. Strengthening of oncology nursing education and training in Africa in the year of the nurse and midwife: addressing the challenges to improve cancer control in Africa. Ecancermedicalscience [Internet]. 2021 Mar 23 [cited 2022 Oct 19];15:1209. Available from: https://www.ncbi.nlm.nih.gov/pmc/articles/PMC8057781/

16. Challinor JM, Hollis R, Freidank C, Verhoeven C. Educational Needs and Strategies of Pediatric Oncology Nurses in Low- and Middle-Income Countries: An International Society of Pediatric Oncology Pediatric Oncology in Developing Countries Nursing Working Group Initiative. Cancer Nursing [Internet]. 2014 Jul [cited 2022 Oct 19];37(4):E36–47. Available from: https://journals.lww.com/00002820-201407000-00013

17. Day SW, Garcia J, Antillon F, Wilimas JA, McKeon LM, Carty RM, et al. A Sustainable Model for Pediatric Oncology Nursing Education in Low-Income Countries. Pediatr Blood Cancer [Internet]. 2012 Feb [cited 2022 Oct 25];58(2):163–6. Available from: https://www.ncbi.nlm.nih.gov/pmc/articles/PMC4676073/

18. Andersson N, Klang B, Petersson G. Differences in clinical reasoning among nurses working in highly specialised paediatric care. Journal of Clinical Nursing [Internet]. 2012 [cited 2022 Oct 19];21(5–6):870–9. Available from: http://0.onlinelibrary.wiley.com/doi/abs/10.1111/j.1365-2702.2011.03935.x

19. Hockenberry M, Mulemba T, Nedege A, Madumetse K, Higgins J. Distance-Based Education for Nurses Caring for Children With Cancer in Sub-Saharan Africa. J Pediatr Oncol Nurs [Internet]. 2020 Sep [cited 2021 Jul 8];37(5):321–9. Available from: http://journals.sagepub.com/doi/10.1177/1043454220938355

20. Adejumo PO, Akinyemi KF, Anarado AN, Ndikom CM, Ohaeri BM, Okedare OO, et al. State of oncology nursing training and practice in a southwestern Nigerian teaching hospital. Journal of Nursing Education and Practice [Internet]. 2021 May 27 [cited 2022

For peer review only - http://bmjopen.bmj.com/site/about/guidelines.xhtml

Nov 20];11(9):68. Available from: https://www.sciedupress.com/journal/index.php/jnep/article/view/20389

21. Tynjälä P. Perspectives into learning at the workplace. Educational Research Review [Internet]. 2008 Jan [cited 2022 Nov 11];3(2):130–54. Available from: https://linkinghub.elsevier.com/retrieve/pii/S1747938X0800002X

22. Challinor JM. Addressing the global pediatric oncology nursing workforce gap: An overdue imperative. Asia-Pacific Journal of Oncology Nursing [Internet]. 2022 Feb [cited 2023 Aug 31];9(2):77–8. Available from: https://linkinghub.elsevier.com/retrieve/pii/S2347562522000063

23. Mailloux CG. Using The Essentials of Baccalaureate Education for Professional Nursing Practice (2008) as a Framework for Curriculum Revision. Journal of Professional Nursing [Internet]. 2011 Nov [cited 2021 Jul 15];27(6):385–9. Available from: https://linkinghub.elsevier.com/retrieve/pii/S8755722311000469

24. Oermann MH. Curriculum Revision: Making Informed Decisions. Nurse Educator [Internet]. 2019 [cited 2021 Jul 15];44(1):1. Available from: http://journals.lww.com/10.1097/NNE.0000000000000630

25. Uwayezu MG, Nikuze B, Maree JE, Buswell L, Fitch MI. Competencies for Nurses Regarding Psychosocial Care of Patients With Cancer in Africa: An Imperative for Action. JCO Global Oncology [Internet]. 2022 May [cited 2023 Aug 28];(8):e2100240. Available from: https://ascopubs.org/doi/10.1200/GO.21.00240

26. Galassi A, Challinor J. Strengthening the oncology nurse workforce in low-income and middle-income countries. Lancet Oncology [Internet]. 2015 Aug [cited 2022 Nov 23];16(8):887–8. Available from: https://www.proquest.com/docview/1702711806/abstract/C8B2B9281D954432PQ/1

27. Mitema A, Maree L, Young A. Cancer treatment in Africa: the importance of the role of nursing. Ecancermedicalscience [Internet]. 2019 Jul 25 [cited 2022 Nov 11];13:944. Available from: https://www.ncbi.nlm.nih.gov/pmc/articles/PMC6722106/

28. Sullivan CE, Morrissey L, Day SW, Chen Y, Shirey M, Landier W. Predictors of Hospitals' Nonachievement of Baseline Nursing Standards for Pediatric Oncology. Cancer Nursing [Internet]. 2020 Aug [cited 2022 Nov 11];43(4):E197. Available from: https://journals.lww.com/cancernursingonline/Fulltext/2020/07000/Predictors_of_Hospitals__Nonachievement_of.13.aspx

29. Karim S, Sunderji Z, Jalink M, Mohamed S, Mallick I, Msadabwe-Chikuni SC, et al. Oncology training and education initiatives in low and middle income countries: a scoping review. Ecancermedicalscience [Internet]. 2021 Sep 30 [cited 2022 Apr 29];15:1296. Available from: https://www.ncbi.nlm.nih.gov/pmc/articles/PMC8580602/

30. Topperzer MK, Hoffmann M, Roug LI, Larsen HB, Lausen B, Schmiegelow K, et al. Unmet need for interprofessional education in paediatric cancer: a scoping review. Support Care Cancer [Internet]. 2019 [cited 2022 Nov 23];27(10):3627–37. Available from: https://www.ncbi.nlm.nih.gov/pmc/articles/PMC6726701/

31. Silva A, Galica J, Woo K, Ross-White A, Luctkar-Flude M. The use of simulation-based education in cancer care: a scoping review protocol. International Journal of Healthcare

For peer review only - http://bmjopen.bmj.com/site/about/guidelines.xhtml

Simulation [Internet]. 00:00:00.0 [cited 2022 Nov 11];1(2):37–41. Available from: https://www.ijohs.com/article/doi/10.54531/dlvs9567

32. Bryan G, Kelly P, Chesters H, Franklin J, Griffiths H, Langton L, et al. Access to and experience of education for children and adolescents with cancer: a scoping review protocol. Syst Rev [Internet]. 2021 Dec [cited 2022 Apr 29];10(1):167. Available from: https://systematicreviewsjournal.biomedcentral.com/articles/10.1186/s13643-021-01723-4

33. Peters MDJ, Marnie C, Tricco AC, Pollock D, Munn Z, Alexander L, et al. Updated methodological guidance for the conduct of scoping reviews. JBI Evidence Synthesis [Internet]. 2020 Oct [cited 2023 Jan 31];18(10):2119–26. Available from: https://journals.lww.com/10.11124/JBIES-20-00167

34. Piper C. System for the Unified Management, Assessment, and Review of Information (SUMARI). J Med Libr Assoc [Internet]. 2019 Oct [cited 2022 Nov 20];107(4):634–6. Available from: https://www.ncbi.nlm.nih.gov/pmc/articles/PMC6774554/

For peer review only

Appendix 1: Search Strategies used across databases

Database	Items Searched	Results
Scopus	1. TITLE-ABS-KEY ("continuous professional development" OR "short learning programme" OR "in-service training" OR "nursing education" OR "service training")	118,479
	2. neoplasm* OR "paediatric cancer" OR "childhood cancer"	3,185,488
	3. TITLE-ABS-KEY ("pediatric oncology nursing" OR "paediatric oncology" OR "oncology nursing" OR "paediatric oncology nursing")	17,449
	4. (TITLE-ABS-KEY ("continuous professional development" OR "short learning programme" OR "in-service training" OR "nursing education" OR "service training")) AND (neoplasm OR "paediatric cancer"' OR "childhood cancer") AND (TITLE-ABS-KEY ("pediatric oncology nursing" OR "paediatric oncology" OR "oncology nursing" OR "paediatric oncology nursing")	**152**
PubMed	1. ((((neoplasm [MeSH Terms]) OR ('paediatric cancer'[Title/Abstract])) OR ('childhood cancer'[Title/Abstract])	1,271,317
	2. ((((pediatric oncology nursing [Title/Abstract]) OR (Paediatric oncology [Title/Abstract])) OR (oncology nursing [MeSH Terms])) OR (paediatric oncology nursing [Title/Abstract])	3082
	3. ((((continuous professional development [Title/Abstract]) OR (short learning programme [Title/Abstract])) OR (Inservice Training [MeSH Terms])) OR (education, nursing [MeSH Terms]	26,276
	4. #1 AND #2 AND #3	**102**
Embase	1. 'neoplasm'/exp OR neoplasm OR 'paediatric cancer' OR 'childhood cancer'/exp OR 'childhood cancer'	6,021,304
	2. 'pediatric oncology nursing'	973
	3. 'nursing education' OR 'in service training' OR 'continuing education' OR 'continuous professional development'	148,334
	4. #1 AND #2 AND #3	**92**
Dimensions	1. "Paediatric oncology" OR "Paediatric oncology nursing" AND "childhood cancer" OR "Paediatric	**36**

For peer review only - http://bmjopen.bmj.com/site/about/guidelines.xhtml

	cancer" OR neoplasms AND "nursing education" OR "in service training" OR "continuing education" OR "continuous professional development"	
CINAHL	1. SU neoplasms OR SU paediatric cancer OR SU childhood cancer OR SU paediatric oncology	1,007
	2. TX pediatric oncology nursing OR TX paediatric oncology OR TX oncology nursing OR TX pediatric oncology nursing	138
	3. SU continuous professional development OR TX short learning program* OR TX in-service training OR TX in service training for nurses OR SU nursing education OR TX service training	1854
	4. S1 AND S2 AND S3	**1216**
St. Jude Children's Research Hospital	1. paediatric oncology nursing OR childhood cancer nursing AND continuing education OR orientation training OR educational programmes OR in-service training	**916**

For peer review only

Appendix 2: A draft data extraction tool

	Studies			
**Item**	**1**	**2**	**3**	**4 etc.**
Author				
Year				
Journal				
Country				
Host site/ institution				
Collaborations				
Partner country				
Partner institution				
Location of training				
Project title				
Target population				
Theory content				
Practical areas				
Programme delivery methods				
Programme duration				
Mode of assessment				
Cultural/ contextual issues				
Theoretical framework				

For peer review only

PRISMA-P Checklist

Section and topic	Item no.	Checklist item
**Administrative information**		
Title:	Paediatric oncology nursing education and training programmes: A scoping review protocol	
Identification	1a	This is a protocol of a scoping review
Update	1b	This protocol is for a new scoping review
Registration	2	The protocol was registered with the Open Science Framework with registration link/number Osf.io/x3q4h<br>Page 10. Line 309
**Authors:**		
Contact	3a	Page 1, 10 and 11<br>Maureen D. Majamanda[1,2,3], mdmajamanda@kuhes.ac.mw<br>Felix Chisoni[4], fchisoni@kuhes.ac.mw<br>Apatsa Selemani [2,4,5], aselemani@kuhes.ac.mw<br>Irene Kearns[3], irene.kearns@wits.ac.za<br>Johanna E. Maree[3] lize.maree@wits.ac.za<br>[1]School of Nursing, Kamuzu University of Health Sciences, Malawi.<br>[2]Consortium for Advanced Research Training in Africa, Nairobi, Kenya.

For peer review only - http://bmjopen.bmj.com/site/about/guidelines.xhtml

		[3] Department of Nursing Education, School of Therapeutic Sciences, Faculty of Health Sciences, University of the Witwatersrand. South Africa.<br>[4]Library Department, Kamuzu University of Health Sciences, Malawi.<br>[5]School of Public Health, Faculty of Health Sciences, University of the Witwatersrand. South Africa.<br><br>Corresponding author<br>Maureen D.Majamanda<br>Kamuzu University of Health Sciences, School of Nursing, Chipatala Avenue, P.O Box 415, Blantyre, Malawi.
Contributions	3b	Page 10<br>The review was drafted and designed by MDM. The revision was performed by FC, AS, IK, JEM. All authors approved the final scoping review protocol and agreed on the submission for publication in a peer reviewed scientific journal.
Amendments	4	This is a new protocol
**Support:**		
Sources	5a	The review has not been funded

For peer review only - http://bmjopen.bmj.com/site/about/guidelines.xhtml

Sponsor	5b	This work is supported by the Consortium for Advanced Research Training in Africa (CARTA). CARTA is jointly led by the African Population and Health Research Center and the University of the Witwatersrand and funded by the Carnegie Corporation of New York (Grant No. G-19-57145), Sida (Grant No:**54100113**), Uppsala Monitoring Center, Norwegian Agency for Development Cooperation (Norad), and by the Wellcome Trust [reference no. 107768/Z/15/Z] and the UK Foreign, Commonwealth & Development Office, with support from the Developing Excellence in Leadership, Training and Science in Africa (DELTAS Africa) programme. The statements made and views expressed are solely the responsibility of the Fellow. For the purpose of open access, the author has applied a CC BY public copyright license to any Author Accepted Manuscript version arising from this submission."
Role of sponsor or funder	5c	None
**Introduction**		
Rationale	6	Page 2

For peer review only

		This scoping review aims to describe the existing literature focusing on paediatric oncology nursing education and training programmes; to map the content, delivery methods, duration and mode of assessment.
Objectives	7	Page 5 and 6<br><br>The current scoping review aims at answering the following research questions:<br><br>1. What is the content of the paediatric oncology nursing education and training programmes?<br>2. What delivery methods did the paediatric oncology nursing education and training programmes use?<br>3. What was the duration of the paediatric oncology nursing education and training programmes?<br>4. How were the paediatric oncology nursing education and training programmes assessed?
**Methods**		
Eligibility criteria	8	Page 6

For peer review only - http://bmjopen.bmj.com/site/about/guidelines.xhtml

		This review will use the following inclusion criteria using the Participant, Context, and Concept (PCC) mnemonic:<br><br>***Participant***<br>This review will focus on articles about paediatric oncology nursing education and training programmes. Information on paediatric oncology nursing only will be extracted from literature that combine education and training programmes for adult and paediatric oncology nursing. For studies that focused on multidisciplinary teams, we will extract information for nurses only.<br><br>***Concept***<br>The review will include articles that describe a paediatric oncology nursing education and training programme. The programme can be continued education, orientation training or short learning programme. Short learning programme in this review is defined as any education and training programme offered for a period of two hours to six months. This definition has been developed by the reviewers based on preliminary search and own experience with short learning

For peer review only - http://bmjopen.bmj.com/site/about/guidelines.xhtml

For peer review only

		programmes. Information to be extracted will include content, delivery methods, duration and mode of assessment.<br><br>***Context***<br>The review will include articles published in peer reviewed scientific journals and grey literature from the ProQuest Dissertation & Theses Global, paediatric oncology reports and guidelines. Articles written in English language from 2012 to 2022 will be included in the review. A 10-year period is broad enough to provide recent evidence in paediatric oncology nursing. Articles from both LMICs and HICs will be included.
Information sources	9	Page 8<br><br>The search will be conducted in the following databases: CINAHL, Dimensions, Embase, PubMed, Scopus and Education Resources Information Center (ERIC). The search will aim to identify published articles from peer reviewed journals. The search will include grey literature from the ProQuest Dissertation and Theses Global, paediatric oncology reports and guidelines as well as paediatric oncology national and international society websites. Reference lists of relevant articles will be screened to identify additional articles.

For peer review only - http://bmjopen.bmj.com/site/about/guidelines.xhtml

		Articles from 2012 to 2022
Search strategy	10	This has been included as appendix 1 and Table 1
**Study records:**		
Data management	11a	Page 8<br><br>Bibliographic results of the searches from all the databases including reference lists will be downloaded and uploaded into Zotero Reference Management Software for deduplication, then uploaded into Joanna Briggs Institute System for the Unified Management, Assessment, and Review of Information (JBI SUMARI) for title and abstract screening. JBI SUMARI is a web-based review application that supports the steps involved in the review process from drafting a protocol, selection of studies, critical appraisal, data extraction and synthesis. It also allows one to manage review teams and contributors to the review [34].
Selection process	11b	Page 8<br>A two-stage standardised screening process will be employed to evaluate eligibility of the articles. Two independent reviewers (MDM, FC) will first screen the title and abstracts of the captured articles. Full text articles of the included studies will then be downloaded.

For peer review only - http://bmjopen.bmj.com/site/about/guidelines.xhtml

		Disagreements between the two reviewers will be discussed with the other three reviewers (AS, LM, IK) and final decision will be made.
Data collection process	11c	Page 8<br>Two independent reviewers will extract data from included articles. This will be performed using a data extraction tool developed by MDM. The data extraction tool will be tested on a sample of papers to determine its practicality prior to the data extraction process.<br><br>A draft data extraction tool is provided (see Appendix 3). The draft data extraction tool will be modified and revised as necessary during the data extraction process. Modifications will be detailed in the full scoping review. Any disagreements that arise between the reviewers will be resolved through discussion or with a third reviewer.<br>Assessment of quality of included articles will not be performed as it is not within the scope of this review.
Data items	12	Page 8<br>The data to be extracted will include: author, year, journal, country, host site/ institution, collaborations, location of training, project title, theory content, practical areas, cultural/ contextual issues, programme

For peer review only

		delivery methods, programme duration, mode of assessment and theoretical framework underpinning the training programme.
Outcomes and prioritization	13	Page 8<br>The data to be extracted will include: author, year, journal, country, host site/ institution, collaborations, location of training, project title, theory content, practical areas, cultural/ contextual issues, programme delivery methods, programme duration, mode of assessment and theoretical framework underpinning the training programme.
Risk of bias in individual studies	14	This is not applicable for this scoping review.
Data synthesis	15a	This is not applicable for this scoping review
	15b	This is not applicable for this scoping review
	15c	This is not applicable for this scoping review
	15d	Page 9<br>Content analysis using basic coding of data will be performed. The results will be described narratively, and presented using summary of findings table and flow-diagrams.
Meta-bias(es)	16	This is not applicable for this scoping review
Confidence in cumulative evidence	17	This is not applicable for this scoping review

For peer review only - http://bmjopen.bmj.com/site/about/guidelines.xhtml
